# Geometry Optimization with Machine Trained Topological Atoms

**DOI:** 10.1038/s41598-017-12600-3

**Published:** 2017-10-09

**Authors:** François Zielinski, Peter I. Maxwell, Timothy L. Fletcher, Stuart J. Davie, Nicodemo Di Pasquale, Salvatore Cardamone, Matthew J. L. Mills, Paul L. A. Popelier

**Affiliations:** 1 Manchester Institute of Biotechnology (MIB), 131 Princess Street, Manchester, M1 7DN United Kingdom; 2 School of Chemistry, University of Manchester, Oxford Road, Manchester, M13 9PL United Kingdom; 3 Biomass Science and Conversion Technology Department, Sandia National Laboratories, Livermore, CA USA; 4 Present Address: Joint BioEnergy Institute, Emeryville, CA 94608 USA

## Abstract

The geometry optimization of a water molecule with a novel type of energy function called FFLUX is presented, which bypasses the traditional bonded potentials. Instead, topologically-partitioned atomic energies are trained by the machine learning method kriging to predict their IQA atomic energies for a previously unseen molecular geometry. Proof-of-concept that FFLUX’s architecture is suitable for geometry optimization is rigorously demonstrated. It is found that accurate kriging models can optimize 2000 distorted geometries to within 0.28 kJ mol^−1^ of the corresponding *ab initio* energy, and 50% of those to within 0.05 kJ mol^−1^. Kriging models are robust enough to optimize the molecular geometry to sub-noise accuracy, when two thirds of the geometric inputs are outside the training range of that model. Finally, the individual components of the potential energy are analyzed, and chemical intuition is reflected in the independent behavior of the three energy terms ${E}_{{\rm{intra}}}^{{\rm{A}}}$(intra-atomic), ${V}_{{\rm{cl}}}^{\text{AA}\text{'}}$ (electrostatic) and ${V}_{{\rm{x}}}^{\text{AA}\text{'}}$ (exchange), in contrast to standard force fields.

## Introduction

Traditional force fields express energy as a function of the internal coordinates of a chemical system. These potential energy functions are only loosely connected to an underlying quantum mechanical reality, if at all. Typically, the various force field energy contributions each fall into one of two broad categories: bonded (covalent) and non-bonded (non-covalent). Although this may appear a natural and innocent partitioning, the sharp distinction does not properly reflect the complexity of the atomic interactions found in condensed matter. Hydrogen bonding is probably the oldest type of interaction to challenge the artificial distinction between bonded and non-bonded interaction. Indeed, the modern approach of Interacting Quantum Atoms (IQA)^1^, which works with finite-volume topological atoms^2–4^, offers a view of covalency as a sliding scale^5,6^. Despite the built-in, artificial nature of their composite functions, popular force fields preserve the binary approach, and utilize a variety of bonded energy terms (such as bond-stretching, angle-bending, torsional rotation and their cross terms), and non-bonded energy terms (such as van der Waals interactions and point charge electrostatics).

A second major feature of these force fields is that the energy expressions are written as penalty functions. For example, if a given bond takes on its equilibrium bond length then the corresponding bond stretch energy is zero. Any deviations from equilibrium (either by bond compression or elongation) result in a positive energy penalty. The force field thus needs a reference geometry (i.e. the equilibrium geometry). Furthermore, the typical Lennard-Jones potential appearing in the modelling of van der Waals interactions introduces its own reference minimum-energy distances. The electrostatic interaction, which is typically written as a Coulomb interaction between point charges, introduces another reference, namely, that of charges being infinitely far apart.

Here, we use a very different approach, called FFLUX. This method, which was formerly called QCTFF^7^, is one in which atoms endowed with quantum mechanical knowledge^8^ come together to form a molecule. The topological energy partitioning method, IQA, offers a route to accomplish this goal when it is combined with a machine learning method. The latter (in this work) is kriging^9–11^ (or Gaussian process regression), which unlike neural networks or genetic algorithms originated in geostatistics. Kriging is a method of interpolation, giving the best linear unbiased prediction of the intermediate values. In 2009 this method was first used^12^ in combination with topological atoms in work that successfully captured the fluctuation of multipole moments of atoms in water clusters (up to the hexamer) in response to geometrical changes in the clusters. This advance constituted the first application of kriging in the context of intermolecular potentials, soon followed by the careful construction^13^ of interatomic potentials for solid state simulations.

We further developed the aforementioned proof-of-concept, and have demonstrated the applicability of kriged topological atoms in a growing variety of cases, including: water clusters^14,15^, methanol^16^, *N*-methylacetamide^17^, cholesterol^18^, a microhydrated sodium ion^19^, all proteinogenic amino acids^20,21^ including aromatic amino acids^22^, alanine helices^23^, hydrogen-bonded^24^ and weakly bound complexes^25^ (both from the S22 data set), and carbohydrates^26^. This collective work displayed the performance of the kriging models in terms of the accuracy of their energy predictions. For that purpose, we typically showed the cumulative error distribution (the so-called “S-curve”) of the energy of each of the test geometries of the kriged system. As the intersection between the set of training geometries and the set of test geometries is the empty set, this type of validation is external. Over the years, the validation of the kriging models has been very systematic, complete and candid. In this same tradition we now systematically investigate a truly novel type of geometry optimization where a kriging model informs an atom on “how to behave” in the presence of other atoms. Ever since the availability of analytical forces^27^ for kriging models, it has been possible to make nuclei move towards an energy minimum. Herein the first application of this technology is described.

In the current paper we show how the geometry of a water molecule can be optimized without ever using bonded force field potentials. Instead, we let atoms adjust themselves as guided by quantum mechanical energy contributions as defined within IQA, including kinetic, exchange, and Coulomb energies. We focus on the essential, technical points of the method, and on its application. We then report various observations collected from a variety of kriging water models: (i) a statistical assessment of the kriging models’ predictions, (ii) an in-depth look at the optimization performance of the various models and optimizers’ parametrizations, (iii) a robustness test over a large set of starting points, and (iv) chemical insight obtained from the IQA framework in combination with FLUXX.

## Computational Methods

**Figure 1:**
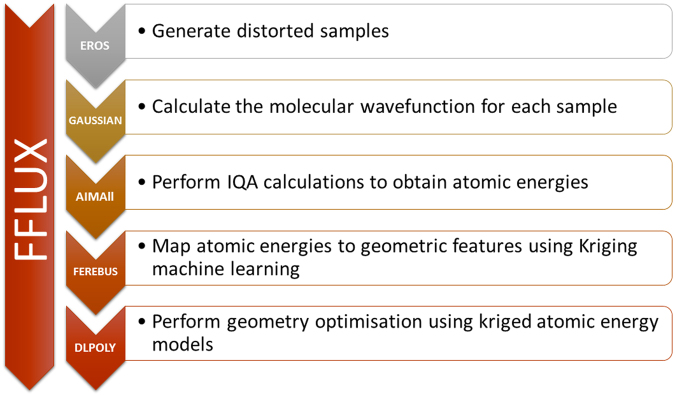
Flowchart of FFLUX’s training (first four steps) and execution (DL_POLY), detailing the programs involved and summaries of their tasks.

There are several components of the FFLUX approach that cannot be discussed in great detail here because of space limitations. Below we provide key references to previous work where these components have been carefully and extensively explained. Training of FFLUX is achieved via a number of in-house and external computer programs, called by an in-house script called GAIA, which controls the construction of a kriging model. A detailed flowchart of GAIA is given in the appendix of reference^22^. GAIA controls thousands of input and output files and allows a user to essentially parameterize FFLUX for any system of interest. The GAIA protocol has five key steps: (1) sampling, (2) *ab initio* calculations, (3) atomic property calculations^28^, (4) kriging model building and (5) validation. Each step is carried out sequentially, with the output of the previous step forming the input for the next step. Before discussing these five steps in turn, we summarize in Fig. 1 the programs used in the construction of kriging models. The first four steps of the GAIA protocol correspond with the first four steps in the diagram of Fig. 1, while the validation step in GAIA usually takes the form of an S-curve (which establishes a kriging model’s energy prediction quality) but can also take the form of an geometry optimization, and later in FFLUX’s development, of an evaluation of a (thermo)dynamic property emerging from a condensed matter molecular dynamics simulation. In the final step of Fig. 1 we mention the molecular dynamics program DL_POLY, which can perform these validations, because it uses FFLUX’s kriging models (in so-called production mode).

### Sampling by normal modes

A single water molecule was geometry optimized at HF/6-31 + G(d,p) using the program GAUSSIAN09^29^ (all default settings, with “*nosymm*” and ”*6D*”). A single-point frequency calculation was then performed to compute the second derivatives of the potential energy (Hessian) at the optimized geometry so that the normal modes of vibration could be determined. As described in Cardamone *et al*.^26^, the optimized geometry can be used as a “seed”, about which one can stochastically sample the molecular normal modes. One is then able to collect an ensemble of such samples, and use them as the input for kriging, as we describe in a later section. This sampling methodology has been implemented in the in-house code EROS. To prevent any unphysical geometries from arising during the sampling process, constraints are placed on any samples included in the ensemble. We require that all bond lengths and valence angles are “distorted” by no more than ±20% of their values in the original “seed” geometry. So, for instance, if a given bond length is 1.0 Å in the “seed” geometry, we constrain the value of the bond length to lie in the range 0.8 Å–1.2 Å for all samples in the ensemble. While the magnitude of distortion is a free parameter, we have found from experience that a value of ±20% allows for an extensive sampling of conformational space, without having to resort to the multi-reference wavefunction techniques required for heavily distorted systems. This procedure was used to generate 2000 water geometries distorted from the geometry-optimized seed.

### *Ab initio* calculations

The wavefunction of each of the 2000 geometries was calculated by GAUSSIAN09 at HF/6-31 + G(d,p). Here we are interested in demonstrating that FFLUX geometry optimization works. Hence we proceed with Hartree-Fock wavefunctions, whose IQA partitioning is simple and well-defined; more advanced wavefunctions will be introduced in subsequent work. Note that the QM minimum wavefunction is never included in the machine learning training sets described below, in order to more strictly test the capability of kriging.

### Atomic property calculations

The IQA method is part of an overall approach, coined^30^ Quantum Chemical Topology (QCT)^31^ in 2003, which is based on the central idea of (gradient) vector field partitioning. This crucial idea lies at the heart of the Quantum Theory of Atom in Molecules (QTAIM)^2,32^, which was the first component of QCT. Recently, QCT has been didactically explained from various angles^3,33,34^. QTAIM (and hence QCT) defines a topological atom, which has a well-defined electronic kinetic energy^35^. This feature is important in the design of a force field with a deep connection to quantum mechanics. IQA partitions a molecule’s energy, ${E}_{{\rm{IQA}}}^{{\rm{Mol}}}$, into a sum of atomic energies, $\sum _{A}{E}_{{\rm{IQA}}}^{{\rm{A}}}$, which in turn are composed of *intra*-atomic and *inter*-atomic energy components,

1 $${E}_{{\rm{IQA}}}^{{\rm{Mol}}}=\sum _{A}{E}_{{\rm{IQA}}}^{{\rm{A}}}=\sum _{A}[{E}_{{\rm{intra}}}^{{\rm{A}}}+\frac{1}{2}\sum _{B\ne A}{V}_{{\rm{inter}}}^{{\rm{AB}}}]=\sum _{A}{E}_{{\rm{intra}}}^{{\rm{A}}}+\frac{1}{2}\sum _{A}\sum _{B\ne A}({V}_{{\rm{cl}}}^{{\rm{AB}}}+{V}_{{\rm{x}}}^{{\rm{AB}}})$$

where A and B represent atoms, and the subscript denotes the type of energy contribution. This equation contains the four types of IQA energy contribution that are relevant to the current study: the overall atomic energy ${E}_{{\rm{IQA}}}^{{\rm{A}}}$, the intra-atomic (or self) energy ${E}_{{\rm{intra}}}^{{\rm{A}}}$, the exchange energy ${V}_{{\rm{x}}}^{{\rm{A}}{\rm{B}}}$, and finally the (classical) Coulomb energy ${V}_{{\rm{cl}}}^{{\rm{AB}}}$. We now briefly explain these primary energy contributions.

The intra-atomic energy ${E}_{{\rm{intra}}}^{{\rm{A}}}$ consists of kinetic, *T,* and potential energy, *V*, contributions:

2 $${E}_{{\rm{intra}}}^{{\rm{A}}}={T}^{{\rm{A}}}+{V}_{{\rm{ee}}}^{{\rm{AA}}}+{V}_{{\rm{en}}}^{{\rm{AA}}}$$

where *T* ^*A*^ represents the kinetic energy of atom *A*, ${V}_{{\rm{en}}}^{{\rm{AA}}}$ is the (attractive) potential energy between the electrons and nucleus of atom *A*, and ${V}_{{\rm{ee}}}^{{\rm{AA}}}$ is the (repulsive) potential energy between the electrons within atom *A*. The latter quantity can be generalized for any atom pair, ${V}_{{\rm{ee}}}^{{\rm{AB}}}$, and further broken down as follows:

3 $${V}_{{\rm{ee}}}^{{\rm{AB}}}={V}_{{\rm{Coul}}}^{{\rm{AB}}}+{V}_{{\rm{x}}}^{{\rm{AB}}}$$

where ‘Coul’ refers to the Coulombic interaction between the electrons and ‘x’ represents the exchange energy. A third term, representing the correlation energy, is missing at Hartree-Fock level. Now that the Coulombic energy has been separated from ${V}_{{\rm{ee}}}^{{\rm{AB}}}$, the classical electrostatic energy ${V}_{{\rm{cl}}}^{{\rm{AB}}}$ can be calculated by including the interaction involving the nucleus of *A* and of *B*,

4 $${V}_{{\rm{cl}}}^{{\rm{AB}}}=({V}_{{\rm{nn}}}^{{\rm{AB}}}+{V}_{{\rm{en}}}^{{\rm{AB}}}+{V}_{{\rm{ne}}}^{{\rm{AB}}})+{V}_{{\rm{Coul}}}^{{\rm{AB}}}$$

Note that the order of the superscripts and subscripts is important because ${V}_{{\rm{en}}}^{{\rm{AB}}}$, for example, refers to the electrons of *A* interacting with the nucleus of *B*, and not the other way around.

Now that the explanation of the primary energy contributions is complete, one more remark needs to be made. A recent FFLUX publication^36^ introduced the use of interatomic energies designated by *AA’* instead of *AB*, a notation employed herein. Here *A’* represents every other atom in the molecular system except *A*. Thus, the notation *AA’* denotes the interatomic energy between an atom *A* and its surrounding environment *A’*, such that

5 $${V}^{AA\text{'}}\cong \sum _{B\ne A}{V}^{AB}$$

where the approximate equal sign is due to the *AA*’ energy being calculated analytically (which is more accurate), but the *AB* energies by quadrature. Finally, the commercial package AIMAll^37^ is used to calculate these energy contributions from the wavefunctions using default parameters, except for the use of AIMAll’s own implementation for computation of the two electron parts of ${V}_{{ee}}^{{AA}}$ (rather than the so-called TWOe integration option).

### Kriging model building

Any machine learning method essentially links a set of inputs (called ”features”) with a set of outputs. Our use of kriging links a set of nuclear coordinates (the features or inputs) with a single output at a time (i.e. one of four possible types of atomic energy). Because the output depends only on the *internal* geometry of the molecule, there are 3*N*-6 features, for a system with *N* atoms. In the case of water there will be three features: two O-H bond lengths and the H-O-H angle. The general definition of features introduces a broader context in which a local axis system, called an atomic local frame (ALF), must be installed on the atom being trained for. Strictly speaking, the axis system is only necessary for outputs that are directional quantities, such as atomic multipole moments, which do not appear in this article. However, the idea of installing an origin at the nuclear position of each atom, one at a time, must be explained here because this installation determines the way the features are constructed.

The *x*-axis of the ALF points from the origin atom to its heaviest bonded neighbour (following the Cahn-Ingold-Prelog convention). The *xy*-plane sweeps out from the *x*-axis toward the second heaviest atom bonded to the origin atom. The origin atom and first and second bonded atoms then determine the *xy-*plane. Subsequently, the *y*-axis is constructed to be orthogonal to the *x*-axis and the *z*-axis orthogonal to both, forming a right-handed axis system. The first three features consist of: (i) the distance between the origin atom (A_1_, 1^st^ atom) and the “*x*-axis-atom” (A_2_, 2^nd^ atom), (ii) the distance between the origin atom (A_1_) and the “*xy*-plane-atom” (A_3_, 3^rd^ atom), and (iii) the angle A_2_-A_1_-A_3_. For oxygen in water, the features are d(OH_1_), d(OH_2_) and α(H_1_OH_2_); for H_1_, they are d(OH_1_), d(H_1_H_2_) and α(OH_1_H_2_); and for H_2_, they are d(OH_2_), d(H_1_H_2_) and α(OH_2_H_1_). For large molecules, the molecular geometry is then converted from Cartesian coordinates in a global frame, to spherical polar coordinates of each remaining atom in the ALF (i.e. those atoms which are not part of the installation of the ALF). Note that each atom in the system acts as an origin for its own ALF, allowing the description of the remaining atoms by a unique (but complete) set of spherical polar coordinates.

Each atom in the system now sees its environment as a set of features (model inputs) and has a set of IQA properties (outputs, one per kriging model) that together make up a single training example for that atom. Since each geometry is a unique training example, and 2000 geometries were sampled, each atom in the system has a list of 2000 training examples (termed a ‘sample set’). GAIA cleans the data through a “scrubbing” process by finding any examples with an AIMAll integration error (measured by L(Ω)) larger than a specified threshold, and removing these examples from the sample set. Any geometry that is incomplete due to removed atoms is then discarded from all atoms’ sample set. Two atomic integration L(Ω)^38,39^ threshold values were used in this work. First, a value of 0.001 au was used for the training sets called “100”, “300” and “500” in the following (which contain 100, 300 and 500 training examples, respectively). Second, a twenty times tighter threshold (of 0.00005 a.u. or 0.13 kJ mol^−1^) was applied for a second training set with 500 examples, termed “T500”. Additionally, a third training set was created, termed “TE500”, which replaces ${V}_{{\rm{cl}}}$, ${V}_{{\rm{xc}}}$, ${E}_{{\rm{intra}}}$ with a single value, namely that of ${E}_{{\rm{IQA}}}$. We have shown before that kriging can successfully^36^ construct a relationship between the various energy contributions and the (geometrical) features.

The kriging method outlined here is based on the treatment of Jones *et al*.^40,41^ and has been explained in much greater detail in our previous work^17^ and also in references^14,42^. Kriging maps the response of an output *ŷ* (an IQA energy) to any given input **x** (set of geometric coordinates),

6 $$\hat{y}({\bf{x}})=\hat{\mu }+\sum _{i=1}^{n}{a}_{i}\cdot \varphi ({\bf{x}}-{{\bf{x}}}^{i})$$

where $\hat{\mu }$ is the estimated global mean of the process, the background value for this output, and *n* is the number of training geometries. The quantity *a*_*i*_ is the *i* ^th^ element of the vector ${\boldsymbol{a}}={{\boldsymbol{R}}}^{-1}({\boldsymbol{y}}-1\hat{\mu })$ where **R** is a matrix of error correlations between training points, and **1** is a column vector of ones. The error from the global term is determined^17^ by the distance between the new input point (**x**) and a known input point (**x** ^i^). The sum of these errors gives the appropriate deviation from the background term and results in the new output, $\hat{y}({\bf{x}})$. An IQA energy has a mean ‘background’ value when considered across many geometries and kriging can map the deviations from the mean in response to geometric changes. The fact that kriging uses the distance between the new input and known inputs is chemically sensible as we can assume that if two geometries are very similar, the IQA energies on the atoms in each geometry are similar as well.

The symmetric correlation matrix **R** consists of the following kernel,

7 $${R}_{ij}=\exp [-\sum _{h=1}^{d}{\theta }_{h}{|{x}_{h}^{i}-{x}_{h}^{j}|}^{{p}_{h}}]$$

where *d* is the number of features, that is, the dimensionality of the input space, which is 3 in the current case study. In general, this value is equal to the number of internal coordinates, i.e. *3N-6*. The correlation between two points in the training data is a function of the distance between the points, along with the kriging hyperparameters **θ** and **p**. These two sets of parameters may both be optimized in order for this correlation to best describe the effect that a move between these two inputs has on the selected output. Note that each dimension (feature) of the kriging problem has its own θ_h_ and *p*_*h*_ value. It has been suggested that *p*_*h*_ can be fixed at 2 for most cases but instead it is optimized alongside θ_h_, because optimizing *p*_*h*_ tends to help the kriging process with small molecules. This process is carried out with an implementation of the particle swarm optimization algorithm, with a log-likelihood objective function in our in-house code FEREBUS^42^. When the kriging training process is complete, a model is created that can be used to predict the IQA properties belonging to an atom when given a previously-unseen geometry. Any remaining data (that is, data that has not already been used to train models) left in the training sets can potentially be used as test examples as the kriging models have no knowledge of these examples.

### Validation

The long-term strategy of FFLUX is one of bottom-up validation. This means that a kriging model is first assessed by the accuracy of its energy predictions. This is done via an S-curve, which will be explained in the Results Section. The next level of validation is based on prediction of a minimum energy geometry, which is achieved for the first time with FFLUX in this article. In order to achieve this aim, we need the analytical force that applies on each nucleus.. The next level of validation, which will be achieved in future work, is that of structure and dynamics obtained from a molecular dynamics (MD) simulation. This highest level of validation will appear in future work on kriged topological atoms, which covers the case of polarizable atoms if kriging trains for atomic dipole moments (and higher rank moments). In the case of non-polarizable topological atoms, their high-rank multipolar potentials have previously been tested against experiment for radial distribution functions and thermodynamic properties, for liquid water^43,44^, liquid imidazole^45^ and aqueous imidazole solutions^46^.

### Atomic forces

For each Kriged quantity, i.e. IQA atomic energy, first derivatives are computed through adapted routines from earlier work dedicated to Kriged multipolar electrostatic interaction^27^. In the present case, in the absence of multipole moments, the reported mathematical framework essentially simplifies itself to:

8 $${F}_{i}^{{\rm{\Omega }}}=-\sum _{A}(\frac{\partial {E}_{{\rm{intra}}}^{{\rm{A}}}}{\partial {\alpha }_{i}^{{\rm{\Omega }}}}+\frac{1}{2}\frac{\partial {V}_{cl}^{AA\text{'}}}{\partial {\alpha }_{i}^{{\rm{\Omega }}}}+\frac{1}{2}\frac{\partial {V}_{xc}^{AA\text{'}}}{\partial {\alpha }_{i}^{{\rm{\Omega }}}})$$

where we differentiated with respect to the *i* ^*th*^ Cartesian coordinate α (*i* = 1, 2, 3 referring to *x*, *y*, or *z*, respectively) expressed in the global frame. Each *E* and *V* quantity is represented by a kriging model or sum thereof. Note that a kriging model is expressed with respect to internal coordinates,

9 $$E=\hat{y}({\bf{x}})=\mu +\sum _{j=1}^{n}{a}_{j}\exp (-\sum _{h=1}^{d}{\theta }_{h}{|{x}_{h}^{j}-{x}_{h}|}^{{p}_{h}})$$

where *E* refers to any of the four types of energy, for any atom or pair of atoms, **x** = {*x*_*h*_; *h* = 1, 2, …, *d*} is a given set of features or internal coordinates for which the energy needs to be predicted, and *d* and *n* again refer to the number of features and training examples, respectively. The derivative of this term with respect to an internal coordinate is given by:

10 $$\frac{\partial E}{\partial {x}_{k}}=\sum _{j=1}^{n}{a}_{j}{\delta }_{jk}(-{\theta }_{k}\,{p}_{k}|{x}_{k}^{j}-{x}_{k}{|}^{{p}_{k}-1})\exp (-\sum _{h=1}^{d}{\theta }_{h}|{x}_{h}^{j}-{x}_{h}{|}^{{p}_{h}})$$

where the cusp in the derivative of the absolute difference at ${x}_{k}^{j}={x}_{k}$ is dealt with by defining

11 $${\delta }_{jk}=\{\begin{array}{c}\,\,\,1\,{\rm{if}}\,{x}_{k}^{j}-{x}_{k}\le 0\\ \quad \,\\ -1\,{\rm{if}}\,{x}_{k}^{j}-{x}_{k} > 0\end{array}$$

The chain rule of differentiation serves as a bridge between Cartesian and internal coordinates or,

12 $$\frac{\partial E({\bf{x}})}{\partial {\alpha }_{i}^{{\rm{\Omega }}}}=\sum _{h=1}^{d}\frac{\partial E({\bf{x}})}{\partial {x}_{h}}\frac{\partial {x}_{h}}{\partial {\alpha }_{i}^{{\rm{\Omega }}}}$$

The kriging derivatives of the various energies can then be directly summed into atomic forces, once converted from the ALF to the global Cartesian frame by applying the chain rule.

### Geometry Optimization

DL_POLY v4.05^47^ was chosen to host adapted code from the group’s kriging prediction engine into a prototype dedicated to both proof-of-concept and design explorations. Several of the host software’s capabilities, parallelism in particular, have been deactivated to facilitate the design and implementation of our method. The current local code demonstrates the viability of our method for gradient-based optimization techniques on water.

The current implementation chose to keep the new modules as self-contained as possible in order to minimize intervention into DL_POLY’s core. By doing so, no changes had to be made to the Verlet integration or the optimization routines, which enabled seamless operation of the MD software. The 0 K (zero Kelvin) optimizer is equivalent to a molecular dynamics run set at minimal temperature, with the particles’ velocities reset to zero between each step. In practice, an atom strictly moves along the forces to which it is subjected. Similar to a MD run, such an optimization then relies on the duration and number of timesteps as parameters. Long timesteps mean the optimum would be reached faster, at the risk of overshooting or oscillating around a narrow and deep minimum, while short timesteps would converge more slowly and risk being stuck in shallow and spurious local minima appearing in an undulating PES.

The conjugate gradient (CG) method proceeds by following the direction of a guess vector until the system’s energy rises, at which point a new vector is computed, as a conjugate of the current last-guess. As a first parameter, the length of CG steps is based on the timestep length in DL_POLY’s implementation. The latter also provides three different convergence criteria to stop the optimization process when satisfied: energy, forces, or displacement (“distance”). So far, only the last one is compatible with our kriging engine. Further details on the optimizers can be found in DL_POLY’s user manual.

## Results

### Kriging model quality (S-curves)

**Figure 2:**
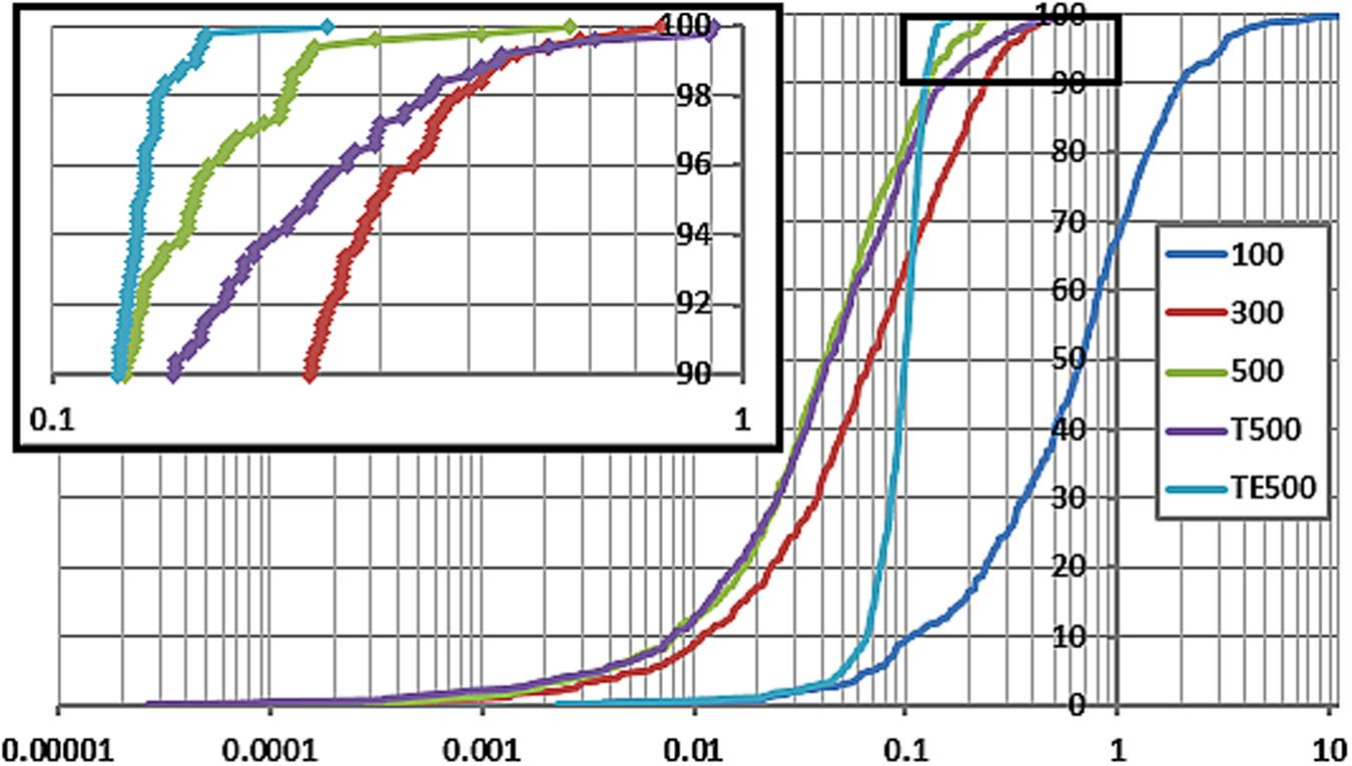
S-curves for the 100, 300, 500, T500 and TE500 water models described using the three energies given in Eqn. 1 (${E}_{{\rm{intra}}}^{{\rm{A}}}$, ${V}_{{\rm{cl}}}^{\text{AA}\text{'}}$ and ${V}_{{\rm{x}}}^{\text{AA}\text{'}}$). The label “T” stands for the tighter scrubbing threshold of 0.00005 Hartrees, while “TE” stands for this tight model using single total atomic energies, ${E}_{{\rm{IQA}}}^{{\rm{A}}}$.

The optimized geometry of water (hereafter referred to as the “QM minimum”) was distorted using the program EROS with a maximal ±20% bond stretch and angle control parameter. The resultant O-H bond range was 0.754 Å ≤ x ≤ 1.132 Å, and the H-O-H angle range 85.70° ≤ x ≤ 128.54°. The sample set had a molecular energy range of 201.6 kJ mol^−1^. The program FEREBUS was used to obtain the molecular models. The quality of the five molecular models (100, 300, 500, T500 and TE500) is illustrated in the S-curves in Fig. 2, supplemented by the statistics given in Table 1. S-curves plot the prediction error (*x*-axis) of each test prediction as a function of the number of test points (in our case 500, *y*-axis) so that each increment of 100%/500 = 0.2% on the *y*-axis represents a test point. Plotting test predictions on an S-curve allows a thorough inspection of a kriging model’s quality. Within an S-curve, hallmarks of a good model are: (i) a steep gradient over a wide range centred at the curve’s inflection point, (ii) being positioned as much as possible to the left, and (iii) a short ‘tail’ at the top (near 100%). The tail refers to the general portion of the curve where the highest errors are seen on the approach to the final point at 100%.

**Table 1:** Statistical analysis of the performance of the five water kriging models.

Measure	Model				
	100	300	500	T500	TE500
Test Set Energy Range	194.8	199.6	199.6	179.2	179.2
Training Set Energy Range	181.0	197.5	199.4	188.7	188.7
Maximum Error	16.6	0.8	0.6	0.9	0.3
Mean Absolute Error (MAE)	1.00	0.10	0.06	0.07	0.10
Prediction % Error	0.51	0.05	0.03	0.04	0.05

All energies are in kJ mol^−1^.

Three observations follow from the S-curves: (i) increasing the training set size from 100 to 300, and again to 500, incrementally moves the curve to the left, resulting in lower Mean Absolute Errors (MAEs), (ii) using a tighter scrubbing threshold *T* (0.00005 Hartrees instead of 0.001 Hartrees) showed little effect on the position of the S-curve, and (iii) kriging the single ${E}_{{\rm{IQA}}}^{{\rm{A}}}$ atomic energy instead of each of ${E}_{{\rm{intra}}}^{{\rm{A}}}$, ${V}_{{\rm{cl}}}^{\text{AA}\text{'}}$ and ${V}_{{\rm{x}}}^{\text{AA}\text{'}}$ dramatically increased the gradient of the S-curve and shortened the tail. The statistics in Table 1 show us that model TE500 has a smaller range of errors, but a slightly higher MAE (0.10 kJ mol^−1^) compared to either 500 (0.06 kJ mol^−1^) or T500 (0.07 kJ mol^−1^). However, with the exception of the 100 model, all models performed very well, having very low molecular energy errors throughout.

The first section in the Supplementary Information shows an extensive cross-validation analysis showing the adequacy of the 300 water model to capture the behavior of the system.

### Optimization Runs

**Figure 3:**
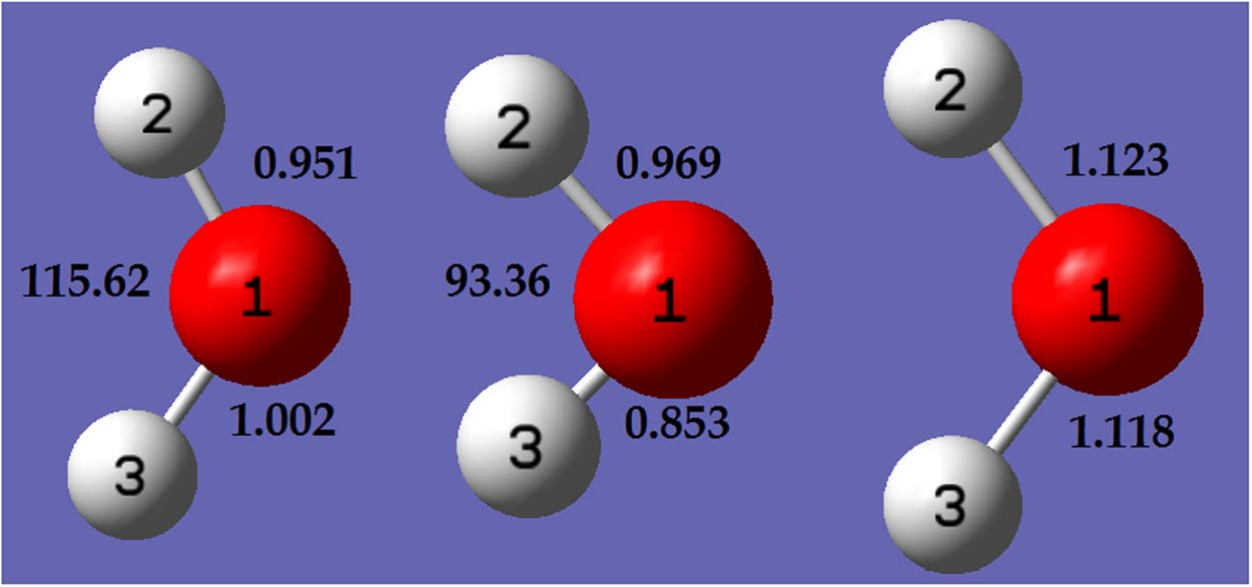
SP1 (left, +15.05 kJ mol^−1^), SP2 (middle, +47.97 kJ mol^−1^) and SP3 (right, +126.18 kJ mol^−1^) water geometries. Bond distances are in Å, and bond angles in degrees.

Having ensured that the generated models are of good quality, the investigation now moves onto their application within the geometry optimization study. From the total sample set, three test samples were chosen as *starting points (SP)* for the initialization of DL_POLY’s geometry optimization run. The three starting points are referred to as SP1, SP2 and SP3, and their relative molecular energies are, respectively, +15.05 kJ mol^−1^, +47.97 kJ mol^−1^ and +126.18 kJ mol^−1^ above that of the QM minimum. Selecting three starting points allows us to investigate each individually, but also to compare and contrast the resulting energies and geometries from each. The three SPs are selected to represent an incrementally more challenging task (from SP1 over SP2 to SP3). However, the geometries of each starting point (illustrated in Fig. 3) also feature three significantly different H-O-H angles of 115.62°, 106.3° and 93.36°, and also three quite different O-H bond length combinations. The increasing molecular energy of each starting geometry, along with three very different bond angles and bond length combinations (which notably feature no symmetry), ensure that the starting points begin their optimization trajectory from significantly different regions of conformational space. The described approach ensures a thorough assessment of each model.

For our preliminary exploration we select two different parameter sets for each of the two optimization methods. The 0 K optimization algorithm was run for 5000 steps with timesteps of 1 fs (parameter setting 1 or “Set 1” in short) and 0.5 fs (“Set 2”), while CG uses 1 fs timesteps with a distance convergence criterion of 10^−5^ Å (“Set 3”) and 10^−6^ Å (“Set 4”). In total, four parameter sets are employed within this investigation

Finally, the kriged PES can be analyzed through a comparison of the geometric features of the optimized water molecules. Should the models have a similar kriged PES, the energies and geometries should show a similar optimization evolution, when starting from the same point. As a first step, here we will limit ourselves to quantitative comparisons on the optimized molecules only. Inconsistent results indicate that the respective PESs of the models are not so similar in the region of the optimal solution. From different SPs, any variation within the resulting energies and geometries within the same model will indicate an undulating PES(in which a trajectory can become trapped in a local minimum).

Table 2 summarizes the molecular optimization energy results for each SP, for all five molecular models. The QM energy (−199,620.00 kJ mol^−1^) is used as the reference energy for all Δ*E* values, calculated as: *ΔE* = *[Final Molecular Energy* − *QM Energy]*. For each parameter setting (i.e. “Set”), the final optimized geometry energy is given along with the corresponding Δ*E*. We now discuss, in turn, four observations.

**Table 2:** SP1, SP2 and SP3 water ${E}_{{\rm{IQA}}}^{{\rm{Mol}}}$ optimization results for each model (100, 300, 500, T500 and TE500).

Model:	100		300		500		T500		TE500	
QM Energy	−199 620.00									
SP1	Energy/kJmol^−1^	ΔE	Energy/kJmol^−1^	ΔE	Energy/kJmol^−1^	ΔE	Energy/kJmol^−1^	ΔE	Energy/kJmol^−1^	ΔE
SP Energy	−199 604.95	15.05	−199 604.95	15.05	−199 604.95	15.05	−199 604.95	15.05	−199 604.95	15.05
Set 1	−199 619.89	0.11	−199 619.88	0.12	−199 619.99	0.01	−199 619.96	0.04	−199 619.86	0.14
Set 2	−199 619.89	0.11	−199 619.88	0.12	−199 619.99	0.01	−199 619.91	0.08	−199 619.86	0.14
Set 3	−199 621.42	−1.42	−199 619.86	0.14	−199 619.96	0.03	−199,619.93	0.06	−199 619.81	0.19
Set 4	−199 621.42	−1.42	−199 619.86	0.14	−199 091.34	—^**a**^	−199,619.93	0.06	−199 619.81	0.19
**SP2**	**Energy**/**kJmol** ^**−1**^	**ΔE**	**Energy**/**kJmol** ^**−1**^	**ΔE**	**Energy**/**kJmol** ^**−1**^	**ΔE**	**Energy**/**kJmol** ^**−1**^	**ΔE**	**Energy**/**kJmol** ^**−1**^	**ΔE**
SP Energy	−199 572.03	47.97	−199 572.03	47.97	−199 572.03	47.97	−199 572.03	47.97	−199 572.03	47.97
Set 1	−199 621.60	−1.60	−199 619.87	0.13	−199 619.99	0.01	−199 620.00	0.00	−199 619.86	0.14
Set 2	−199 620.19	−0.20	−199 619.87	0.13	−199 619.99	0.01	−199 619.96	0.04	−199 619.86	0.14
Set 3	−199 621.40	−1.40	−199 619.84	0.15	−199 619.72	0.28	−199 619.82	0.17	−199 619.79	0.20
Set 4	−199 621.40	−1.40	−199 619.84	0.15	−199 619.72	0.28	−199 619.82	0.17	−199 619.79	0.20
**SP3**	**Energy**/**kJmol** ^−**1**^	**ΔE**	**Energy**/**kJmol** ^**−1**^	**ΔE**	**Energy**/**kJmol** ^**−1**^		**Energy**/**kJmol** ^**−1**^	**ΔE**	**Energy**/**kJmol** ^**−1**^	**ΔE**
SP Energy	−199 493.81	126.18	−199 493.81	126.18	−199 493.81	126.18	−199 493.81	126.18	−199 493.81	126.18
Set 1	−199 621.58	−1.58	−199 619.99	0.00	−199 620.06	−0.06	−199 619.97	0.03	−199 619.86	0.14
Set 2	−199 621.58	−1.58	−199 619.88	0.12	−199 619.99	0.01	−199 619.95	0.04	−199 619.86	0.14
Set 3	−199 620.52	−0.53	−199 619.85	0.14	−199 619.87	0.12	−199 619.83	0.16	−199 619.80	0.20
Set 4	−199 620.52	−0.53	−199 619.85	0.14	−199 619.87	0.12	−199 619.83	0.16	−199 619.80	0.20

QM energy. Set 1: 0 K run for 5000 steps with time step of 1 fs; Set 2: same as Set 1 but 0.5 fs; Set 3: CG with 1 fs and 10^−5^ Å as convergence threshold; Set 4: same as Set 3 but convergence threshold at 10^−6^ Å.

^a^The minimum was never reached for reasons described in the main text (“fourth observation”). Δ*E* is the energy difference between the molecule’s optimized energy and its.

The first observation is that all the optimizations have indeed run successfully with the exception of one (SP1, parameter set 4, for model 500 – details discussed shortly). However, before any further analysis, proof-of-concept has been shown: QCT atoms dressed up with IQA atomic energies and converted into kriging models, indeed are sufficient to obtain atomic forces suitable for molecular geometry optimization.

The second observation is that for all parameter sets (with only the above exception), the Δ*E* of the final geometry is ≤ ±1.6 kJ mol^−1^. In fact, most cases are ≤ ±0.2 kJ mol^−1^. Remarkably, the lowest Δ*E* reported is <0.01 kJ mol^−1^. However, final Δ*E*’s smaller than the model’s MAE (typically within ±0.1 kJ mol^−1^), are within the *accuracy threshold* of our approach. Also, the atomic integration implemented in AIMAll introduces energy noise that typically does not enable us to recover the QM energy closer than within ±0.1 kJ mol^−1^ (or 0.00005 a.u.). With the above in mind, it is remarkable to observe the kriging predictions performing so well. The same energetic minimum is being reached consistently for most SPs, within the same molecular model. Hence, no spurious local minima significantly corrupt the kriging PESs, which still appear unimodal. This observation can be made *across* models too but now returning more variation in the values of the energy minimum reached. Moving from the 100 model, over the 300 model to the 500 model, shows that denser sampling converges closer to the *ab initio* minimum. Note that there is pressure to keep the number of training examples to a minimum because of the computational cost of generating atomic energies. Hence, going beyond 500 cannot be justified, especially given the already excellent results obtained with 500. In summary, our aim is to build a kriging model with the smallest possible training set size, compatible with the accuracy we need for the problem considered.

We also see the abovementioned consistent convergence when operating both above and below the accuracy threshold. For example, in the TE500 model, all six 0 K optimizations (two across each of SP1, SP2 and SP3) all converge to a geometry with the same energy (Δ*E* = +0.14 kJ mol^−1^), above the accuracy limit (±0.1 kJ mol^−1^). For the 500 model we see this consistency (reaching Δ*E* = +0.04 kJ mol^−1^, below the accuracy threshold of ±0.06 kJ mol^−1^) for five of the six 0 K parameter settings. The respective geometries reached for the TE500 and 500 models, are not only energetically the same, but also geometrically, as shown in Table 3, which reports the final geometries of parameter Set 1.

**Table 3:**
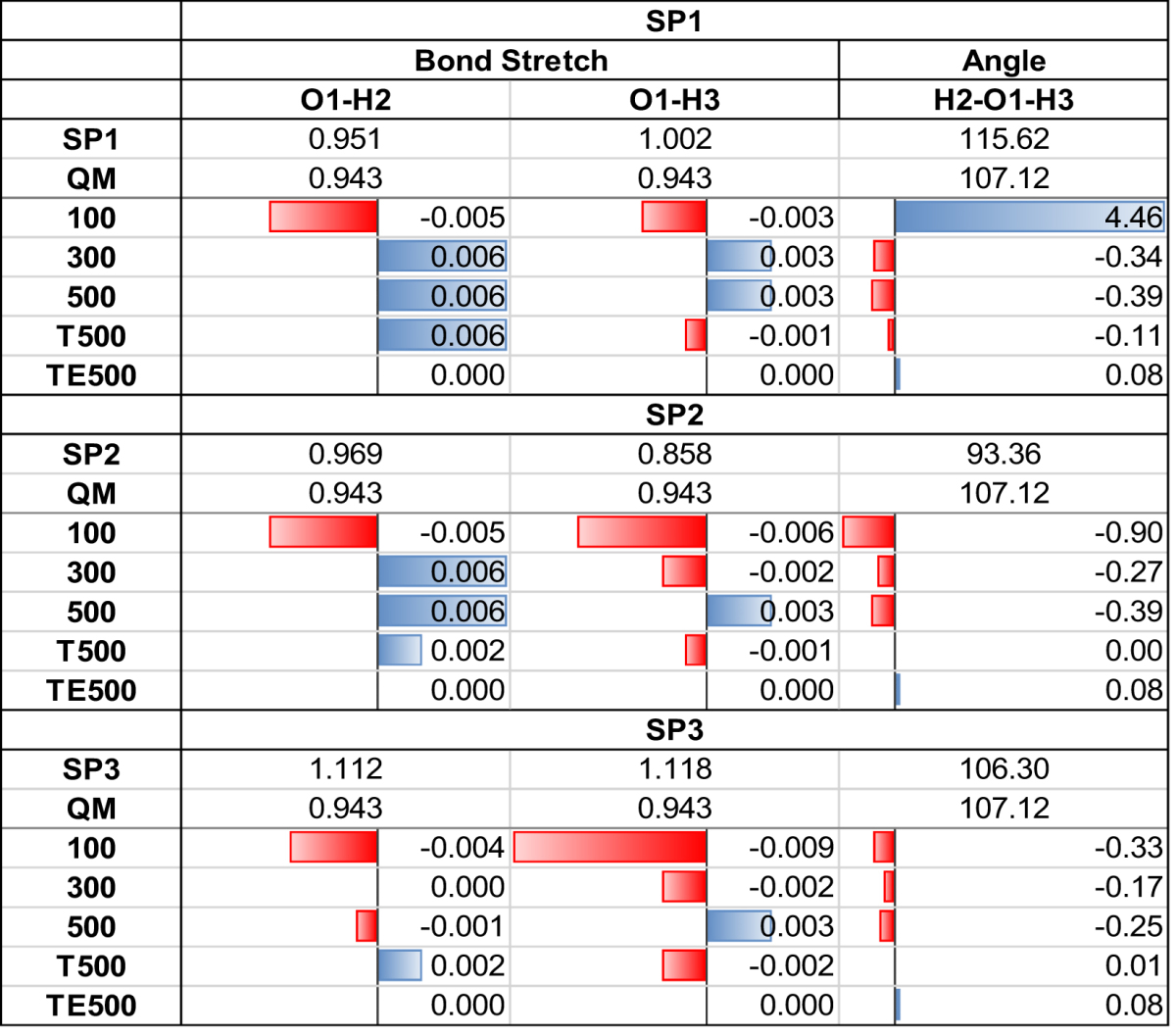
Water’s optimized geometrical data from each starting point (SP1, SP2 and SP3) using the five models with parameter Set 1 throughout. Optimized values are reported as relative to the QM, i.e. bond distances and angles are plotted as “relative data” bars where red indicates a lower value, blue a higher value. The magnitude of each bar is marked by its length, normalized using all resulting bond distances across all three SPs. The largest bar (red, SP3, 100 model) is set to one unit of length. The angles are treated similarly, with the unit length bar being “blue, SP1, 100 model”.

The third observation is that the 0 K optimizations consistently perform better than the CG optimizations, where “better” equates to a lower Δ*E*. Throughout all 15 datasets (5 models × 3 SPs), there is only one example where both CG optimizations perform better than the two 0 K models (100 Model, SP3). The superior accuracy of 0 K was expected, given this method’s design to follow the trajectory of a very low temperature simulation continuously until a chosen number of steps are reached. However, CG places more emphasis on reaching the minimum of the model more quickly, through a reduced number of steps and stopping when a convergence criterion is met. Indeed, although the use of CG has reached a slightly poorer molecular energy minimum geometry than 0 K, is the results are still good. Within the optimization community, the reduced accuracy of CG is generally accepted in favour of a fast calculation. Hence, CG’s firm grounding as a common optimization algorithm, and 0 K’s relative obscurity. For our investigation, 0 K is an undoubtedly useful algorithm for diagnostic purposes and proof-of-concept.

The fourth observation is that “Set 4” fails for SP1 for the “500” training as a result of the trajectory of the optimization never meeting the distance convergence criteria. Such behavior is an indication that the PES modelled in the IQA model is not smooth enough to reach a solution. At some point, the energy gradient of the model may cause a step in an incorrect direction of conformational space. Should such an event lead to the geometry ‘escaping’ the training range far enough to result in the kriging training correlation vanishing, then the geometry is considered to be in the flat “no man’s land” that exists outside of the PES. For the SP1-Set 4 example, the molecular energy fluctuates between a good prediction and a poor prediction as the optimizer attempts to improve it. Eventually the model predicted a point far outside the training range, from which the trajectory failed to recover. A sensible hypothesis is that the PES is not accurate enough within that specific region of the conformational space. The lack of accuracy in the PES is likely either due to nearby points with high integration errors; to the fact that the training set used is not able to correctly describe the PES; or a mix of these two. If the problem is identified in the training set, the poor description of the PES could be due to an insufficient number of training points or a poor sampling of the conformational space. It is very difficult to tell *a priori* which is the main source of error and the more effective ways used to reduce this problem include (i) the use of more training points, (ii) considering more accurate training points, (iii) a better distribution of training points in conformational space, (iv) a combination of the three solutions presented before. Alternatively, completing the optimization with a less strict distance convergence criterion (as in “Set 3”) is a solution that would not involve the modification of the model.

**Figure 4:**
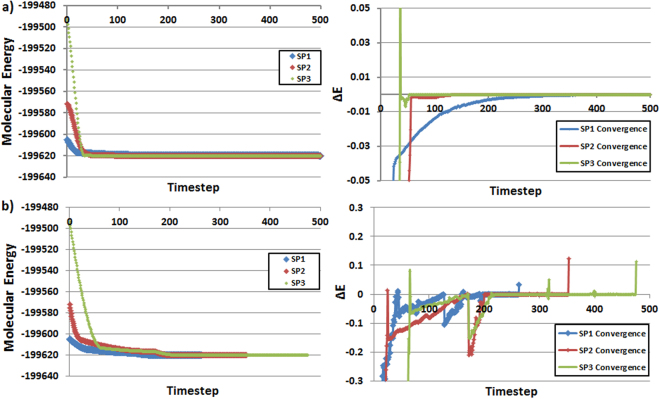
T500 molecular model geometry optimization trajectory steps with SP1 (blue), SP2 (red) and SP3 (green) starting points: (**a**) Set 1 (0 K and 1 fs timestep) truncated at 500 steps where the energy fluctuation is <0.0001 kJ mol^−1^ and (**b**) Set 3 (GC and 1 fs timestep) with no truncation. The *x*-axis marks the timestep number. In the left panels, the *y*-axes denote molecular energy; in the right panels the y-axes denote Δ*E* (current energy – previous energy). All energies are in kJ mol^−1^.

Having analyzed the energy of each final timestep, we now analyse the energy evolution trajectory. Because we observe consistently low energy Δ*E* values for the T500 model, these energy trajectories should provide a good example of the behaviour that one can expect from an accurate kriging water model. Figure 4 shows the energy trajectory of the optimization using Set 1 (0 K and 1 fs) and Set 3 (CG and 10^−5^ Å convergence) for each starting point. The left panels show a difference between the smooth trajectory of the 0 K algorithm (top) and the jagged trajectory expected from CG (bottom). This behaviour is amplified in the right-hand magnified plots. The magnified plots show the convergence for each SP through monitoring the energy differences between successive timesteps. The plots on the right of Fig. 4 define Δ*E* as [*current system energy* − *previous system energy*]. Note that the convergence plot for CG always ends with a peak, caused by the CG algorithm being forced to predict a geometry of higher energy because the landscape does not offer any further minimizing solutions. The final point on each CG plot merely shows this final step. The penultimate step is then treated as the final optimized solution. The 0 K plots are truncated to 500 out of the 5000 completed timesteps since the molecular energy does not fluctuate by more than 0.0001 kJ mol^−1^ following this point. Interestingly, the CG runs almost reach energy convergence on a similar time scale to 0 K. The lack of a clear difference between the number of timesteps is unexpected but can be explained by the fact that such a molecular system could be too small to really benefit from the CG approach. Soon-to-be published work will expand this approach to larger molecules where the number of time steps required for a successful optimization can be readdressed and confirmed with scaled-up examples.

Finally, Set 1 serves as an example to discuss the geometrical aspect of the optimization results. Table 3 summarizes each model’s geometries optimized using Set 1’s parameters. The quantitative data are augmented by data bars representing the deviation of optimized bonds and angles with respect to the QM values. Unsurprisingly, the 100 model is the worst performing, consistently producing geometries that are the most different from the QM optimum. However, even for the 100 model, the correct bond stretches are reproduced to within 0.01 Å, whereas the H-O-H angle is less accurate. The 300, 500 and T500 models all perform relatively similarly, with bond stretches within 0.007 Å and angles within 0.39°. The TE500 model performs exceptionally well, predicting both bond stretches to within 0.0005 Å and the angles to within 0.09°. Note that the TE500 model is the only model to reproduce a symmetrical final geometry. TE500 also optimizes to almost the exact same geometry for all three starting points (with Δ*E* = 0.14 kJ mol^−1^), with the final angles differing < 0.001°. All other models return different optimized angles for each SP, with some models reporting the same bond stretches across some SPs. The lack of consistency for the bond angle confirms that angular features are less energetically influencing than radial features (bond distances).

The above analysis is presented from a critical point of view in order to properly scrutinize the results. However, as seen from the energy results in Table 2, we are often working within the accuracy threshold, and the error margins seen across all the results presented in Table 3 are very low for all but the 100 model. Thus, like the energy analysis, the geometrical features are proven to optimize to their correct values, within very small error margins.

### Starting from a geometry outside the training set

Here we report on the robustness of the models when the optimization is initialized from starting point geometries (SPs) with *energies* that are *all outside* of the training range. The set of 4 starting points will be referred to as SP-OUT 1, SP-OUT 2, SP-OUT 3* and SP-OUT 4*, which start with the following Δ*E* values: 195.24 (+6.53), 201.15 (+13.45), 300.50 (+111.79) and 592.21 (+403.5) kJ mol^−1^, respectively. The geometrical features of each SP-OUT system may be found in Table 4. The asterix, *, indicates that this starting point contained geometric features outside of the training range, and the bracketed values represent the difference in energy from the maximum of the trained energy range, in this case 188.71 kJ mol^−1^ for the T500 model. It was important that at least some of the geometric features lay within the training set range, otherwise the model would not be expected to perform and produce any relevant results. SP-OUT 4 is the only starting point lacking any geometrical features within the training set range, but is included for comparison. To reiterate, when making predictions, a kriging model will default to the mean value (*μ*) when correlation between the training features and an example point’s features vanish, i.e. when a geometry drifts too far outside of the training range. In the case of water, when one (or two) geometric feature(s) are in this position, the remaining two (or one) feature(s) are responsible for guiding the molecule back to within the operable training range. Currently, it is unknown to what extent this is possible. Should all three geometric features be outside the training range, the model is expected to fail and give a poor final geometry.

**Table 4:**
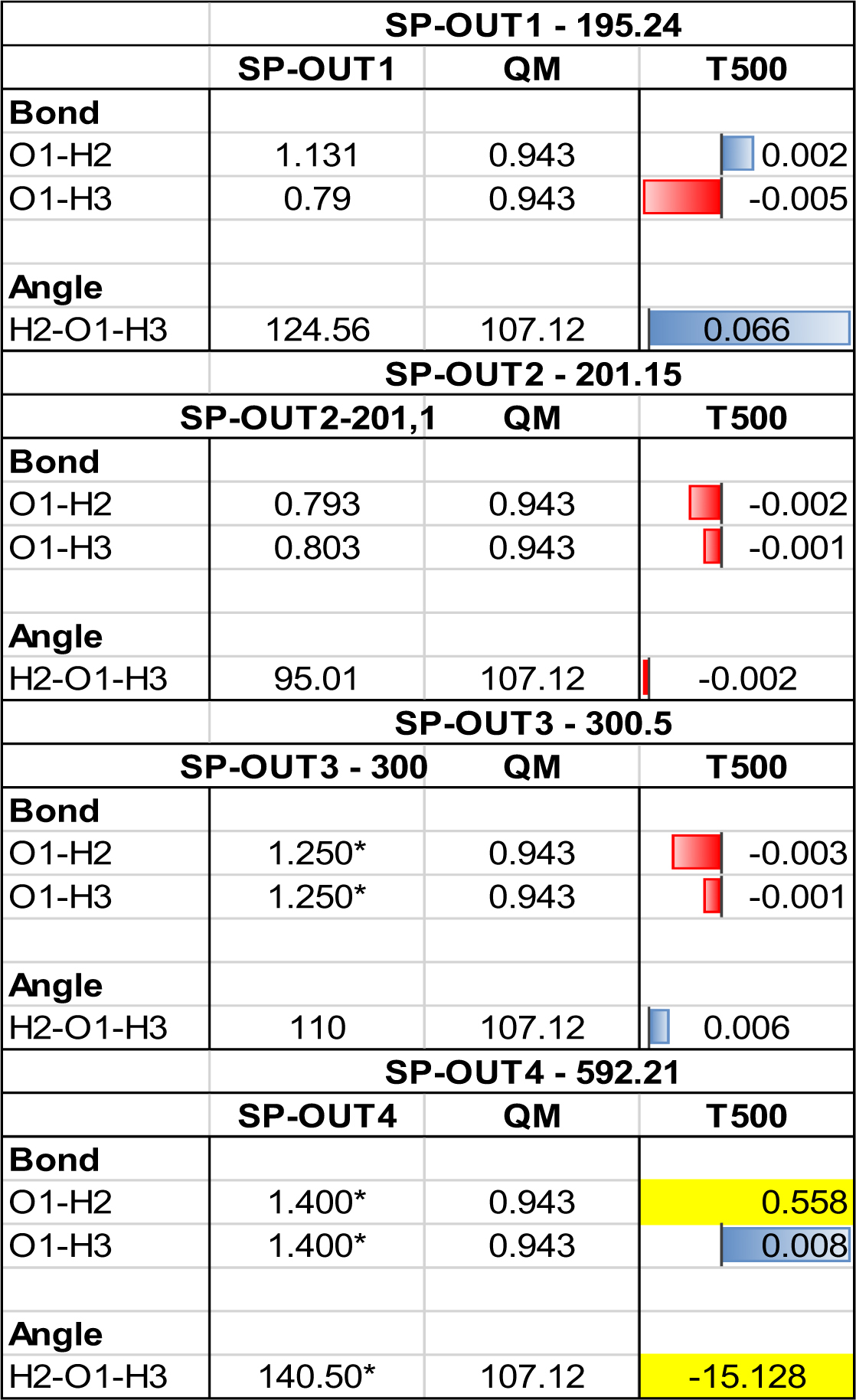
Optimized geometrical data for each of the four SP-OUT runs for the most energetically stable parameter set (Set 1). All runs are completed using the T500 model. Optimized values are reported as relative to the QM, i.e. bond distances and angles are plotted as “relative data” bars where red indicates a lower value, blue a higher value. The magnitude of each bar is marked by its length, normalized using all resulting bond distances across all three SPs. The largest bar (blue, SP-OUT4, O1-H3) is set to one unit of length. The angles are treated similarly, with the unit length bar being “red, SP-OUT1”. Values outside the training range are highlighted in yellow and taken out of the data bars calculations.

Table 5 reports the optimized energies from the four SP-OUT runs, again using the same four parameter sets (Sets 1 to 4). For consistency with Fig. 4, the T500 model is used for this analysis, however, any of the other 500, T500 or TE500 models would have been suitable. Remarkably, the optimizations run successfully for each of SP-OUT1, SP-OUT2 and SP-OUT3 cases, reaching Δ*E* values of less than 0.14 kJ mol^−1^.

**Table 5:** Optimization results from starting points (SP) generated outside (OUT) the training set energy range (called “SP-OUT 1” to “SP-OUT 4”), using the T500 water model.

QM Energy	T500 - Outside 1 – (SP-OUT 1)			T500 - Outside 2 – (SP-OUT 2)		
	−199 620.00			−199 620.00		
	Energy/kJmol^−1^	ΔE/kJmol^−1^	Steps	Energy/kJmol^−1^	ΔE/kJmol^−1^	Steps
SP Energy	−199 424.75	195.24 = 6.53		−199 418.84	201.15 = 12.45	
1	−199 619.89	0.10	5000	−199 619.95	0.05	5000
2	−199 619.86	0.14	5000	−199 619.95	0.05	5000
3	−199 619.86	0.13	360	−199 619.85	0.14	350
4	−199 619.86	0.13	360	−199 619.86	0.13	435
	**T500 - Outside 3 – (SP-OUT 3)**			**T500 - Outside 4 – (SP-OUT 4)**		
SP Energy	−199 319.50	300.50 = 111.79		−199 027.78	592.21 = 403.5	
1	−199 619.94	0.05	5000	−199 482.19	137.81	5000
2	−199 619.95	0.04	5000	−199 482.19	137.81	5000
3	−199 619.96	0.04	563	−199 085.71	534.29	942
4	−199 619.96	0.04	563	−199 085.71	534.29	942

The final geometric data for the runs of Set 1 are given in Table 4, which is analogous to the format of Table 3. Geometrical features appear good, matching the energy optimization for all except SP-OUT4. SP-OUT 4 fails by incorrectly predicting the O1-H2 bond by +0.558 Å and the H-O-H angle by −15.138°. Elongation of the O1-H2 bond causes the O1-H3 bond to shorten and finish with a reasonable final length (only +0.008 Å from the target value). Examples of the geometric trajectory can be found in the Supplementary Information. Figures S2 and S3 depict the fluctuation of the geometric features for SP-OUT 2 Sets 1 and 4 respectively, recovering from outside of the training range and producing a good final geometry. However, Figures S4 (Set 1) and S5 (Set 4) illustrate how such a recovery never occurs with SP-OUT 4. Here, the optimizations eventually terminate with all or some of the final geometric features still not, or never, in the training set range. The behaviour of the final few steps of the optimization in Figure S5 is comparable to that observed in the failed case of [SP1/500 model/“Set 4”] (see Table 2). This behaviour characterizes an evolution outside the operable range of the models.

### Scanning the landscape

**Figure 5:**
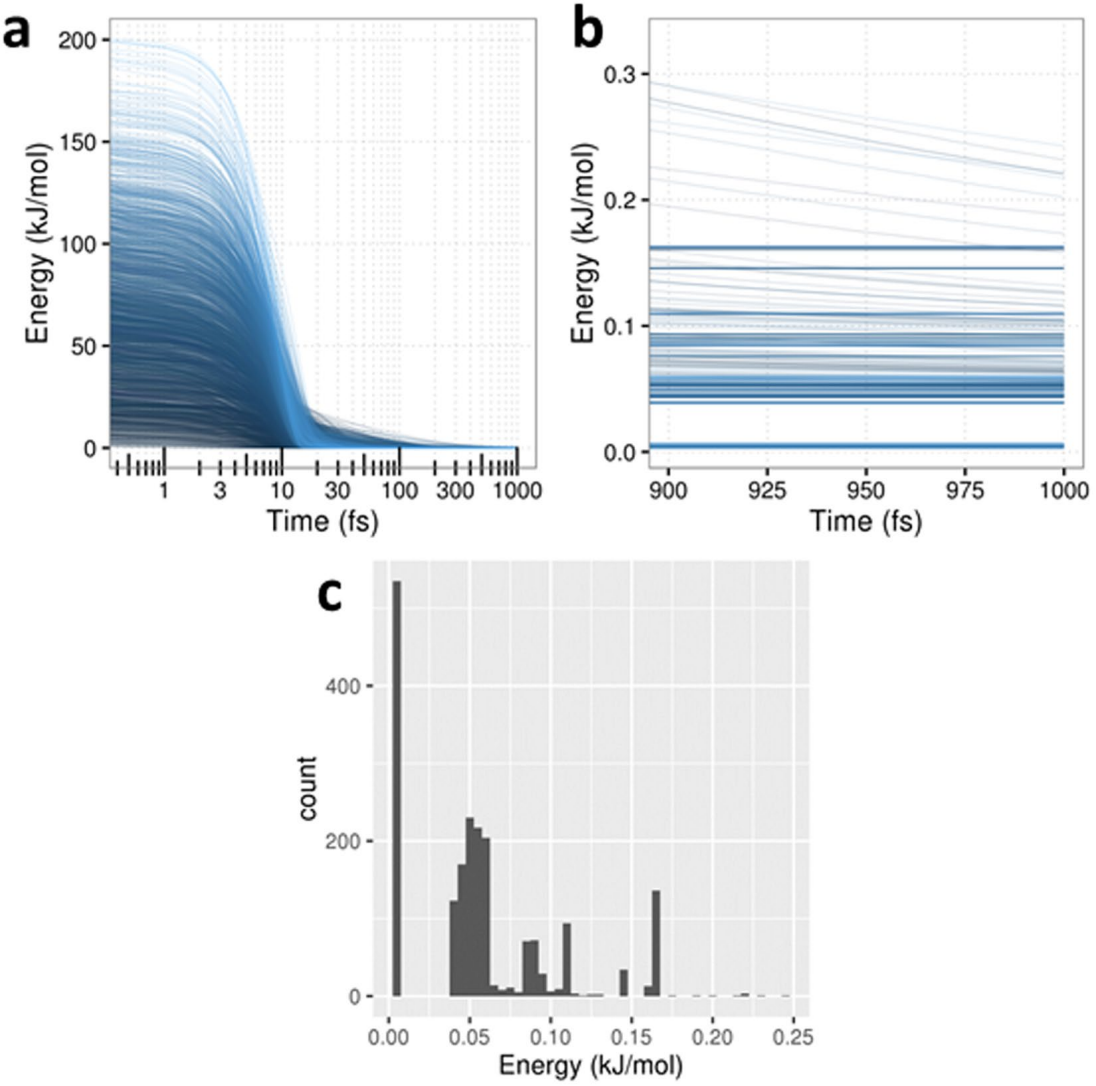
Performance of the T500 model using the 0 K optimization: (**a**) aggregated plot of the molecular energy evolution in time for each of the 2000 starting geometries considered (runs are coloured from dark to light blue allowing tracking); (**b**) magnified energy evolution between the 900^th^ and 1000^th^ timesteps; (**c**) distribution of energies at the 1000^th^ timestep, relative to the ab initio energy.

As a final check of the model’s robustness, a comprehensive analysis was set-up by taking, as starting points, each of the 2000 geometries generated by the distortion method. Optimization runs were carried out for each geometry with the 0 K method (to ensure consistency and proper comparison) for 2000 timesteps of 0.5 fs. From the previous investigations, these parameters were deemed suitable to obtain a bird’s eye view of the set’s general behaviour and detect outliers. Again, the T500 model was selected for this analysis. The energy evolution of every trajectory was then extracted and aggregated, to be plotted in Fig. 5 as differences with respect to the QM minimum. By the 2000^th^ step, the average energy difference reached was 0.056 kJ mol^−1^ (standard deviation: 0.046 kJ mol^−1^, minimum and maximum: 0.003 and 0.24 kJ mol^−1^, respectively).

The generality of the behaviour described earlier for the three individual starting points can also be observed in the top left panel of Fig. 5: every trajectory’s energy monotonically drops within ~15 kJ mol^−1^ of the QM minimum in less than 100 timesteps. The more slowly converging trajectories seem to mostly originate in low-lying energy starting points (darker blue). The final steps, as seen in the top right panel of Fig. 5, reveal several trajectory bundles approaching the expected QM energy minimum by a different amount (again, without clear separation of the trajectories originating from low and high-lying energy starting points). Within the 2000 optimization steps, more than 25% of the set converges within 0.01 kJ mol^−1^, while a bigger portion of the set (~50%) clusters around convergence within 0.05 kJ mol^−1^. Finally, 100% of the set converges within 0.25 kJ mol^−1^. The most probable reason for this behaviour is the inevitable presence of noise (at least with the current method) in the kriging model, caused by the underlying accuracy of the IQA calculations, and leading to small spurious local minima around the global minimum and a less smooth PES surface. In any case, the accuracy threshold we operate within is reasonable enough to consider the whole of the sample set reasonably converging. In order to provide a fuller picture of the convergence behaviour, adequate tools for a proper analysis of the geometry evolution are in development and results will be featured in forthcoming publications featuring a larger variety of systems.

### Individual preferences of the ${E}_{{\rm{intra}}}^{{\rm{A}}}$, ${V}_{{\rm{cl}}}^{\text{AA}\text{'}}$, and ${V}_{{\rm{x}}}^{\text{AA}\text{'}}$ atomic energies

Here we report on the individual tendencies of each of the three IQA energies (${E}_{{\rm{intra}}}^{{\rm{A}}}$, ${V}_{{\rm{x}}}^{\text{AA}\text{'}}$ and ${V}_{{\rm{cl}}}^{\text{AA}\text{'}}$), which are used in four of the five molecular models tested. Observing the ‘ideal’ behaviour of each gives us an insight into the interplay between the knowledgeable topological atoms occurring in each of the optimization runs. Here, we systematically eliminate, in turn, each of the other two energy contributions. First, the ${V}_{{\rm{cl}}}^{\text{AA}\text{'}}$ and ${V}_{{\rm{x}}}^{\text{AA}\text{'}}$ models are switched off to observe only the behaviour of ${E}_{{\rm{intra}}}^{{\rm{A}}}$ in an optimization. Likewise, the next run switches off the ${V}_{{\rm{cl}}}^{\text{AA}\text{'}}$ and ${E}_{{\rm{intra}}}^{{\rm{A}}}$ components to obtain the behaviour of just ${V}_{{\rm{x}}}^{\text{AA}\text{'}}$. The final run completes the analysis by switching off ${V}_{{\rm{x}}}^{\text{AA}\text{'}}$ and ${E}_{{\rm{intra}}}^{{\rm{A}}}$ to observe only the ${V}_{{\rm{cl}}}^{\text{AA}\text{'}}$ behaviour.

**Figure 6:**
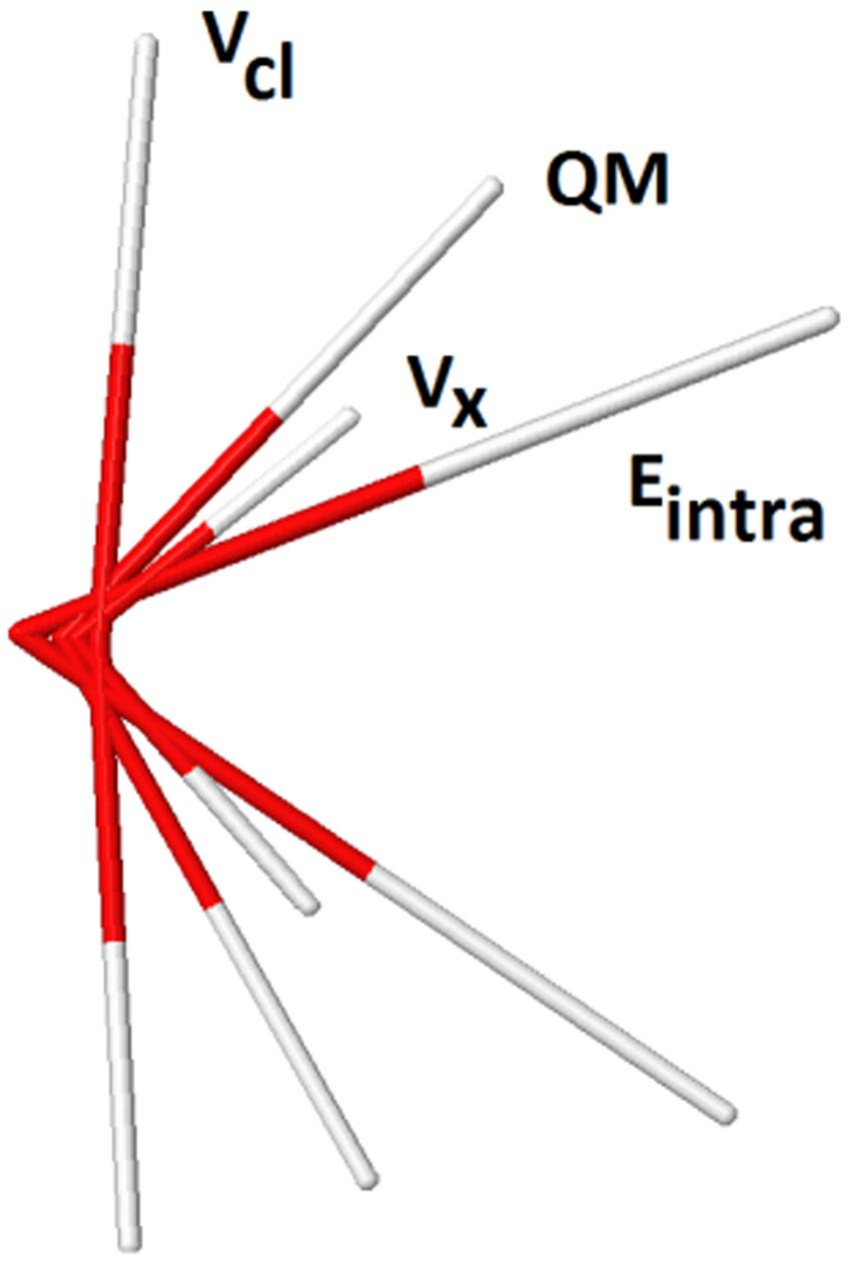
Single-energy optimized water geometries using the individual ${E}_{{\rm{intra}}}^{{\rm{A}}}$, ${V}_{{\rm{x}}}^{\text{AA}\text{'}}$ and ${V}_{{\rm{cl}}}^{\text{AA}\text{'}}$ energies. Initialization geometry is the QM minimum, and the optimizations are performed using the T500 model with parameter Set 1.

Once more, the combination of the T500 model and Set 1 was selected for these examples. At the QM minimum, all three IQA components are optimally balanced: by optimising from the corresponding QM minimum geometry we can then observe each of the individual IQA components’ preferential drift. Figure 6 illustrates the resulting geometries, alongside the QM initialization geometry for reference. Table 6 accompanies the results, analyzing the geometries from Fig. 6 quantitatively, similar to Table 4.

**Table 6:**
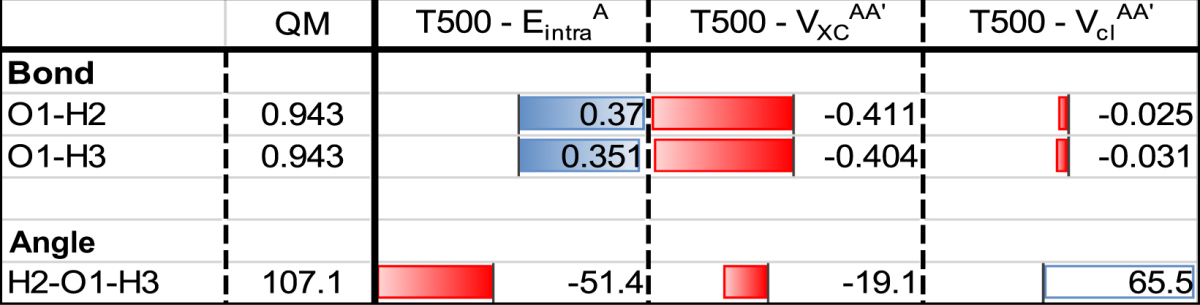
Geometrical data for the single-energy optimized runs, using Set 1 associated to the T500 model. Optimized values are reported as relative to the QM, i.e. the value of [Resulting Feature – QM], and plotted as a relative data bar. Optimized values are reported as relative to the QM, i.e. bond distances and angles are plotted as “relative data” bars where red indicates a lower value, blue a higher value. The magnitude of each bar is marked by its length, normalized using all resulting bond distances across all three SPs. The largest bar (red, ${V}_{{\rm{x}}}^{\text{AA}\text{'}}$, O1-H2) is set to one unit of length. The angles are treated similarly, with the unit length bar being “blue, ${V}_{{\rm{cl}}}^{\text{AA}\text{'}}$”.

First, the Coulomb-only optimization (${V}_{{\rm{cl}}}^{\text{AA}\text{'}}$) slightly compresses the O-H bond lengths but maximizes the H-O-H angle to 172.6°, indicative of the, admittedly very low, electrostatic repulsion between the two hydrogen atoms. Second, the intra-atomic-only optimization (${E}_{{\rm{intra}}}^{{\rm{A}}}$) significantly elongates the O-H bonds and makes the H-O-H angle much more acute (~50°). Unpublished results have shown that ${E}_{{\rm{intra}}}^{{\rm{A}}}$ can generally be correlated with atomic volumes: where ${E}_{{\rm{intra}}}^{{\rm{A}}}$ becomes lower (more stabilized) atomic volume increases and *vice versa*. Hence, elongation of the O-H bond lengths reflects an increase in both the oxygen and hydrogen atomic volumes. However, the H-O-H angle becoming more acute tells us that maximizing the oxygen’s volume must more than compensate for the additional destabilization resulting from the mutual compression of the two hydrogens. Third and finally, the exchange-only optimization (${V}_{{\rm{x}}}^{\text{AA}\text{'}}$) expectedly compresses the O-H bond lengths upon optimization. This compression corresponds to a lowering of the interatomic exchange energy. This energy is numerically dominated by the O-H exchange energy. Thus, the O-H compression corresponds to a strengthening in the covalent bonding in water. However, at some point in the compression the exchange stabilization reaches an extremum, and the bond becomes increasingly unstable again. We can also deduce that the through-space interaction between the H…H atoms must not be significant since it is barely able to make the H-O-H angle more acute (~90°).

The geometries presented in this section should not be overinterpreted, as they are driven outside of the training range (in “no-man’s-land”) and are likely to converge due to the loss of kriging correlation: these analyses are relevant for the geometric drift tendencies only.

The analysis in this section reminds us how there is not only a complex interplay between the type of atomic energies used within the optimization, but also a balance being reached between the atoms when only a single type of IQA energy is used. The interpretation of significance^48^ of each IQA energy could benefit from the perspective provided by such optimizations, at least at the level of chemical intuition, as it gives some insight about where each energy component “pushes” the molecule to go toward. No such analysis is possible for the TE500 model, for which the earlier results (see Section 3.2) already illustrate the preferential behaviour of the ${E}_{{\rm{IQA}}}^{{\rm{A}}}$ energy for an atom in this water model.

These results stand in contrast to those that would be made on a traditional force field potential. For a single water molecule, a typical bonded potential would consist of a single harmonic angle-bend and a pair of identically-typed harmonic bond-stretch terms. Turning off one of these terms while maintaining the remaining two will result in a completely unphysical potential. Removing the angle-bend term gives a potential where the H atoms can occupy the same position with no energetic penalty, while removing either bond-stretch term allows the corresponding O-H interatomic distance to take any value. Neither of these altered potentials can provide any physical insight, in stark contrast to the potential described herein, emphasizing the lack of correspondence between standard force field bonded energy terms and their underlying quantum origins.

## Discussion

### Contextualization of FFLUX

A thorough dissection of the force field research literature proves that the FFLUX methodology is unique and indeed novel. This approach goes further than multipolar force fields (such as SIBFA and AMOEBA), which in turn innovate the traditional point charge force fields (such as AMBER).

The work we presented here is a very detailed theory-versus-theory assessment rather than a theory-versus-experiment comparison. This means that we set up a novel computational scheme (FFLUX) that is asked to reproduce, as best as it can, the original quantum mechanical data that it was trained for. We carefully demonstrated a *proof-of-principle*, at some level of theory that is not the best possible because it does not have to be the best possible, that is, for the current purpose of proof-of-principle.

We worked with Hartree-Fock because the IQA energy contributions are very clear in this case. We took a small basis set because it compensates the inherent error introduced by the limitations of the Hartree-Fock *Ansatz*. As long as the proof-of-principle of the machine-taught topological atoms reaching the global energy minimum together is solid we have reached our goal, no matter at which level of theory this is achieved. The current article reports the first ever such study, and this successful proof-of-concept opens an exciting avenue for a host of applications, on larger molecules and complexes, eventually reaching condensed matter simulations via a rigorous bottom-up research program.

Comparing to other traditional force fields such as AMBER or CHARMM is also moot at this stage. The geometry of a single water molecule in a force field method can be optimized by simply looking up the values for the two reference bond lengths and the one reference angle in the appropriate parameter set. Note that there will be no non-bonded interactions in a standard force field for a single water. These values are almost always chosen by comparison to quantum chemical results anyway; how well a traditional force field describes a single water molecule is of very limited interest. Because of the novelty of the FFLUX method there is already enough to explain on a single water molecule. This system illustrates in an “uncluttered” way how much parameterization effort is already involved. This article raises points that are salient to proving that the method can be used in much more complicated applications (which are the subject of future publications).

We do not intend to ignore the vital question of how a “force field makes contact with experiment”. Of course the long-term goal is to make reliable predictions that are experimentally verifiable. More excitingly, we *eventually* intend to use FFLUX in the area of nucleation, which experiment cannot probe in its very early stage. This is an example where FFLUX’s reliability will be crucial.

In terms of comparison with experiment at this current early stage, the relevant experimental data for a single water molecule are then the 0 K geometry (and potentially its vibrational frequencies), since the geometry is what we aim to compute. However, this geometry is actually not known experimentally: bond lengths are never known as (quantum mechanical) r_e_ values but as any one of derived (i.e. treated) bond lengths (e.g. r_z_ or r_g_ or r_α_ …). In our work we use the Hartree-Fock method as a surrogate for experiment, being straightforwardly (if expensively) replaceable with any other quantum mechanical method in the construction of FFLUX models. This successful and safe strategy defers the question of comparison to experiment to later applications where, for example, liquid density can be evaluated from simulation and directly compared to experiment.

Our overall strategy has always been bottom-up: start from small systems (such as a single water molecule) and then upscale. In the current proof-of-concept stage it is important and indeed sufficient to compare FFLUX with first-principles (i.e. quantum mechanical) data, which is what is done in this paper. In later work we will carry out simulations on liquid water (as we have done with rigid body water molecules, equipped with multipolar electrostatics). We will then, as we have done in our previous publications, compare the computed structure and dynamics with neutron diffraction data, and kinetic and thermodynamic quantities (e.g. self-diffusion coefficient, isothermal expansion coefficient, heat capacity at constant pressure etc.) with measured values.

Compared to a traditional force field, a FFLUX model will always take more computational time but then the former provides less information than FFLUX; indeed, one should not compare profoundly dissimilar objects. FFLUX is a force field that “sees the electrons”, which traditional force fields do not. As demonstrated in the last part of the article, FFLUX is aware of the internal energy of an atom (i.e. intra-atomic), the electrostatic interaction energy and the exchange energy. These are quantum mechanical data that originate from the wave function itself. Traditional force fields do not contain this information. Moreover, FFLUX also stores atomic multipole information (not active in the optimization of water because of divergence of the multipole expansion but the electrostatic interaction is covered by the IQA term *V*_*cl*_ anyway), and also stores their polarization more importantly, which is absent in traditional force fields. Again, this extra information adds to the cost of FFLUX.

It has been clear from the start of the FFLUX project, that FFLUX will be a more expensive force field computationally but, at the beginning of the FFLUX project many years ago, we aimed for computers of the near future, which now exist. Each year passing enables more expensive calculations to become feasible, and thus the reliability and accuracy of FFLUX will increasingly benefit the systems that are currently only within the application radius of traditional force fields.

Building up a database of transferable models will be a computationally expensive task but will remain a one-off, not burdening the user who wants to be shielded from this activity. However, there is nothing stopping users adding to a database of models, much like the PDB or CSD. Building models (more specifically, obtaining the IQA-QM data) is the expensive stage. However, the high transferability of our topological atoms has already proven to reduce the necessary workload.

CPU timings for a single water optimization can be reported at this stage although the code has not been optimized yet. Without compiler optimisation and in debug mode on a single Intel® Core™ i5-2410M CPU@2.30 GHz processor, DL_POLY takes, for 2000 steps (of the iterative geometry optimisation) and 500 training examples, using all three energies, 1.92 seconds, or about one millisecond per step. This result is very encouraging because FFLUX already defeats the original *ab initio* calculation by at least three orders of magnitude (and this is just for Hartree-Fock, which is a cheap *ab initio* method), prior to any source code optimization having taken place.

Finally, we point out that in terms of accuracy the method relies on much more representative physics than a harmonic force field.

### Final Considerations in connection with Future Work

Looking at the nature of the FFLUX approach and its future brings up five topics that benefit from some extra comment at this point in time.

First, many-body effects often feature in force field discussions, particularly in the treatment of liquid water in terms of (long range) perturbation theory. While focusing on water clusters, the concept of many-body effects determines to what extent a water trimer for example, can be described, energetically, by three water dimers. We appreciate this type of analysis but it does not directly affect the IQA analysis that we use in FFLUX. The key point is that all IQA quantities are always extracted from the full wave function, involving all water molecules in the cluster. In other words, the electron density is obtained through the Self-Consistent-Field (SCF) procedure, which relaxes all the orbitals with respect to one another, and thereby automatically accounts for many-body effects. We also note that FFLUX incorporates polarization through kriging models for atomic multipole moments responding to their precise environment. Unlike other polarizable force fields, FFLUX does not invoke and a SCF procedure *during* the molecular dynamics production run. The present method aims, first and foremost, at improving the realism of molecular modelling and bringing it closer to quantum mechanics, which is known to have an even higher computational cost. However, the model offers optimisation opportunities, e.g. in terms of mass-parallelism, with the prospect of reaching quasi-linear scaling on distributed hardware.

Secondly, there are advantages and disadvantages to FFLUX. Attractive features encompass (i) the near *ab initio* accuracy without having to carry out the *ab initio* calculation in the “production phase”, (ii) much faster performance than that of an *ab initio* calculation, especially if the latter is carried out with a high level of theory, (iii) robust chemical insight, physically rooted and free from assumptions. For example, one can find out *why* an energy minimum exists in terms of the balance between intra-atomic self-energy, electrostatic and exchange(-correlation) energies, (iv) no need for a penetration correction, (v) no need for damping functions (because there is no polarisation catastrophe), (vi) FFLUX is well grounded in the literature with precise answers regarding its various aspects, (vii) the topological partitioning is parameter-free and reference-free, (viii) diffuse Gaussian functions are not problematic, and finally (ix) Kriging handles high-dimensional feature spaces well with a relatively small number of data points. Amongst the current disadvantages one can think of (i) the computational expense of IQA, (ii) the performance of kriging models depending on atomic integration errors, (iii) an improved sample point selection approach to correctly represent the configuration space at hand, and finally (iv) the challenge of defining ALFs in condensed matter, in the presence of significant intramolecular hydrogen bonds or π-π stacking, and when molecules travel over large distances.

Thirdly, in terms of suitability for quantum dynamics simulation, FFLUX can in principle be applied to adiabatic Born-Oppenheimer *ab initio* MD, as it provides the ground state total energy and forces for a chemical system required to evaluate the forces on the nuclei. Whether or not this is to be viewed as quantum dynamics is open to interpretation due to the use of the machine learning method as an intermediary between nuclear coordinates and the total energy. Whilst definitely not a force field method, the described potential is in some sense empirical. Beyond *ab initio* MD, FFLUX is not useful in its current state for non-adiabatic calculations as the system wavefunction is only implicit in the output of the machine learning models. Molecular orbital or electron density information is not produced by the Kriging models; the method shares an inherent ground-state nature with force fields. However, for reactions or systems with small band gaps, one would certainly need to be able to incorporate non-adiabatic effects.

Fourthly, FFLUX can be applied to bond breaking, in principle, including chemical reactions and changes in metal coordination. A kriging model can be taught potentially any behaviour, given the correct data. Note that in this study we use kriging to find relationships between energies and geometries, with the geometries being represented by features. Those features can take any value and be defined as arbitrarily as the user requires. Thus it is not only possible to tackle a problem such as bond breaking, it should require no additional terms or fixes such as those found in several force fields when attempting to model new phenomena.

Fifthly and finally, the number of features in the current work is very small but kriging scales exceptionally well. In the recent past we have tackled over 100 features for larger molecules^18,49^ and molecular clusters while maintaining very good kriging predictions.

## Conclusion

For the first time, atomic kriging models have been “set in motion”, through the associated (analytical) forces. Geometry optimizations have been successfully carried out, yielding energies and geometries in agreement with the QM optimum. While used as the model’s seed, the latter is not part of the training set: the ability of our kriging atomic models to generally reproduce a sampled molecular potential is then fully confirmed.

A variety of kriging models were analyzed and compared: complementing the picture provided by S-curves, optimization stands as a new validation tool, closer to practical purposes and sensitive to gradient prediction errors. As expected, models including more training points yield optimized structures closer to the QM reference, both in energy and geometry. The robustness of the kriging models is demonstrated by their ability to fall back into their optimum even when starting outside of their conformational training range. Initializing the optimization from every generated sample point did not reveal any major spurious minimum or other shortcoming on the potential energy surface, thereby corroborating the kriging method.

Finally, chemical insight provided by the IQA energy decomposition is preserved through our method, where observations consistent with intuition have been made by isolating individual contributions.

Further encouraging results are soon-to-be published, featuring more complex molecules and a deeper analysis of the potential energy surfaces, in particular the forces and the agreement between energy predictions and original QM energy.

## Electronic supplementary material

Supp Info
