# Supplementary material for: Geometry Optimization with Machine Trained Topological Atoms
Source: Sci Rep. 2017 Oct 9;7:12817. doi: 10.1038/s41598-017-12600-3 (PMC5634454; doi:10.1038/s41598-017-12600-3)
Supplement: Supplementary file 1 — Supp Info [file 41598_2017_12600_MOESM1_ESM.pdf]

Supplementary Information

Geometry optimization with machine trained topological atoms

François Zielinski, Peter Maxwell, Tim Fletcher, Stuart Davie, Nicodemo Di Pasquale, Salvatore Cardamone, Matthew Mills and Paul L. A. Popelier*

Figure S1. 10-fold cross-validation for O1, H2 and H3. The different symbols represent different IQA energy components: V_x (red circles), Self (or $E_{\text{intra}}^{\text{A}}$) (black diamonds) and V_{cl} (blue squares).

Figure S2. SP-OUT2 Set 1 with T500 model geometric feature optimization.

Figure S3. SP-OUT2 Set 4 with T500 model geometric feature optimization.

Figure S4. SP-OUT4 Set 1 with T500 model geometric feature optimization.

Figure S5. SP-OUT4 Set 4 with T500 model geometric feature optimization.

The results presented in Tables S1-S3 report the analysis of the kriging models represented in this work. The correlation coefficient q^2 is a quick test[1] to analyse the performance of a predictor model. It has the intuitive property to be (i) equal to zero when a kriging model is no better than the naive predictor, represented by the mean of the process, which we want to approximate (Eq. 6 of the main text), and (ii) equal to 1 when the predictions are equal to the true values. The main consequence is that when q^2 is close to 1 the kriging models capture not only the correct values of the points in the prediction set but we can be reasonably sure that also the variation of these values (i.e. the structure, or the behaviour of the real system) is correctly described. The latter is also confirmed by looking at the other columns in Table S1-3. The maximum error is always smaller than 2 kJ mol^{-1}, which represents a few percentage of the real value of the energy in all the cases considered.

**O1 Vx**					
	q^2	Max abs error kJ mol^{-1}	Mean abs error kJ mol^{-1}	Mean true value kJ mol^{-1}	Mean Predicted Value kJ mol^{-1}
100	0.998	1.73	0.40	-509.94	-509.83
300	0.999	1.15	0.24	-508.36	-508.30
500	0.998	0.87	0.17	-507.06	-507.08

**O1 Self**					
	q^2	Max abs error kJ mol^{-1}	Mean abs error kJ mol^{-1}	Mean true value kJ mol^{-1}	Mean Predicted Value kJ mol^{-1}
100	0.999	5.78	1.320	-195625.37	-195625.05
300	0.999	2.53	0.33	-195611.55	-195611.52
500	0.999	1.39	0.21	-195609.70	-195609.72

**O1 Vcl**					
	q^2	Max abs error kJ mol^{-1}	Mean abs error kJ mol^{-1}	Mean true value kJ mol^{-1}	Mean Predicted Value kJ mol^{-1}
100	0.999	7.48	1.92	-860.53	-861.12
300	0.999	3.96	0.57	-878.37	-878.47
500	0.999	1.95	0.37	-880.42	-880.37

Table S1. From top to bottom, metric analysis for oxygen atom labelled O1.

H2 Vx					
	q^2	Max abs error kJ mol^{-1}	Mean abs error kJ mol^{-1}	Mean true value kJ mol^{-1}	Mean Predicted Value kJ mol^{-1}
100	0.999	0.70	0.030	-256.34	-256.33
300	0.999	0.13	0.0080	-255.14	-255.13
500	0.999	0.10	0.0054	-254.48	-254.48

H2 Self					
	q^2	Max abs error kJ mol^{-1}	Mean abs error kJ mol^{-1}	Mean true value kJ mol^{-1}	Mean Predicted Value kJ mol^{-1}
100	0.999	0.98	0.057	-748.91	-748.91
300	0.999	0.094	0.010	-744.82	-744.82
500	0.999	0.11	0.0074	742.37	742.37

H2 Vcl					
	q^2	Max abs error kJ mol^{-1}	Mean abs error kJ mol^{-1}	Mean true value kJ mol^{-1}	Mean Predicted Value kJ mol^{-1}
100	0.999	1.49	0.063	-259.12	-259.14
300	0.999	0.27	0.017	-264.51	-264.51
500	0.999	0.20	0.018	-267.39	-267.39

Table S2. From top to bottom, metric analysis for oxygen atom labelled O1.

H3 Vx					
	q^2	Max abs error kJ mol^{-1}	Mean abs error kJ mol^{-1}	Mean true value kJ mol^{-1}	Mean Predicted Value kJ mol^{-1}
100	0.999	0.16	0.020	-255.62	-255.62
300	0.999	0.077	0.0066	-255.12	-255.12
500	0.999	0.063	0.0051	-254.53	-254.53

H3 Self					
	q^2	Max abs error kJ mol^{-1}	Mean abs error kJ mol^{-1}	Mean true value kJ mol^{-1}	Mean Predicted Value kJ mol^{-1}
100	0.999	0.30	0.036	-750.75	-750.75
300	0.999	0.074	0.0090	-745.22	-745.22
500	0.999	0.072	0.0070	-744.44	-744.44

H3 Vcl					
	q^2	Max abs error kJ mol^{-1}	Mean abs error kJ mol^{-1}	Mean true value kJ mol^{-1}	Mean Predicted Value kJ mol^{-1}
100	0.999	0.33	0.043	-257.20	-257.20
300	0.999	0.12	0.014	-264.08	-264.07
500	0.999	0.12	0.012	-264.58	-264.58

Table S3. From top to bottom, metric analysis for oxygen atom labelled O1.

We report in Figure 1 (just below) the 10-fold validation for the smallest training sets i.e. 100, 300, 500 training points, for O1, H2 and H3. In 10-fold validation, each set is divided in 10 equally sized subsets. We train a model leaving out one subset at a time, to be used as prediction set. We then calculate the mean squared error (MSE) of this prediction set:

$$MSE_{ij} = (y_{ij} - \hat{y}_{ij})^2 \qquad (1)$$

where y_{ij} is the real value of the property at the i-th point of the j-th fold, while $\hat{y}_{ij}$ is the predicted property at the i-th point of the j-th fold. The cross-validation error is calculated as

$$CV_n = \frac{1}{n}\sum_{i=1}^{10}\sum_{j=1}^{k} MSE_{ij} \qquad (2)$$

where n is the size of the training set.

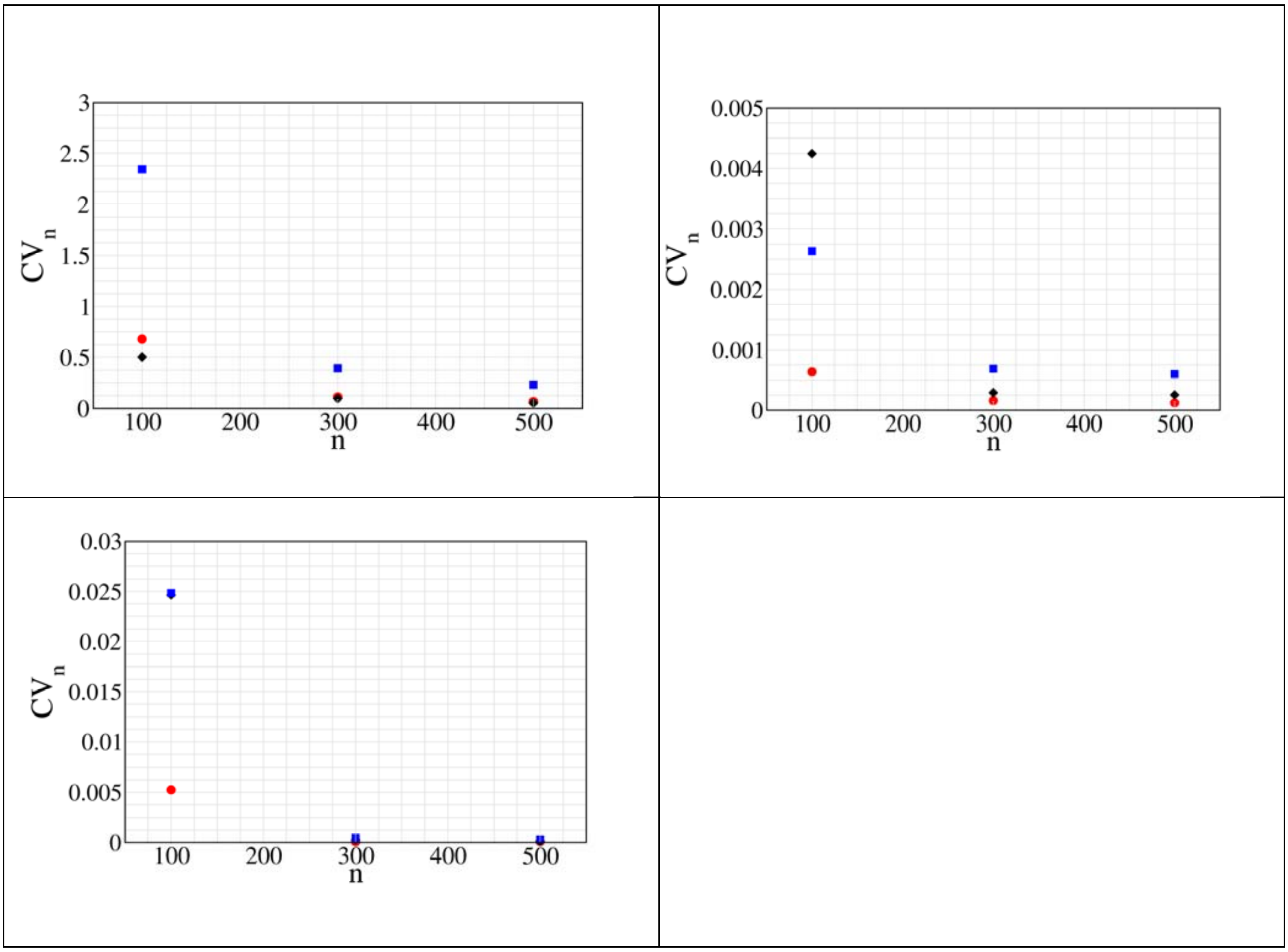

Figure S1. From top to bottom, left to right, 10-fold cross-validation for O1, H2 and H3. The different symbols represent different IQA energy components: V_x (red circles), Self (or E^{A}_{intra}) (black diamonds) and V_{cl} (blue squares).

Although the error is already small in all the cases considered, from the 10-fold validation results it is possible to see that 300 training points are already enough to capture the behaviour of the system. We want to highlight here that all the results we showed in the paper (see Figure S1) are obtained by using an *external* validation set, i.e. a set of points not used in the training, which could be considered a way to validate the predictions when the number of sampled points available is enough to allow the creation of a training set and a validation set[2].

(1) Davie, S. J.; Di Pasquale, N.; Popelier, P. L. A. *J.Comput.Chem.* **2016**, *37*, 2409.
(2) Rasmussen, C. E.; Williams, C. K. I. *Gaussian Processes for Machine Learning.*; The MIT Press: Cambridge, USA, 2006.

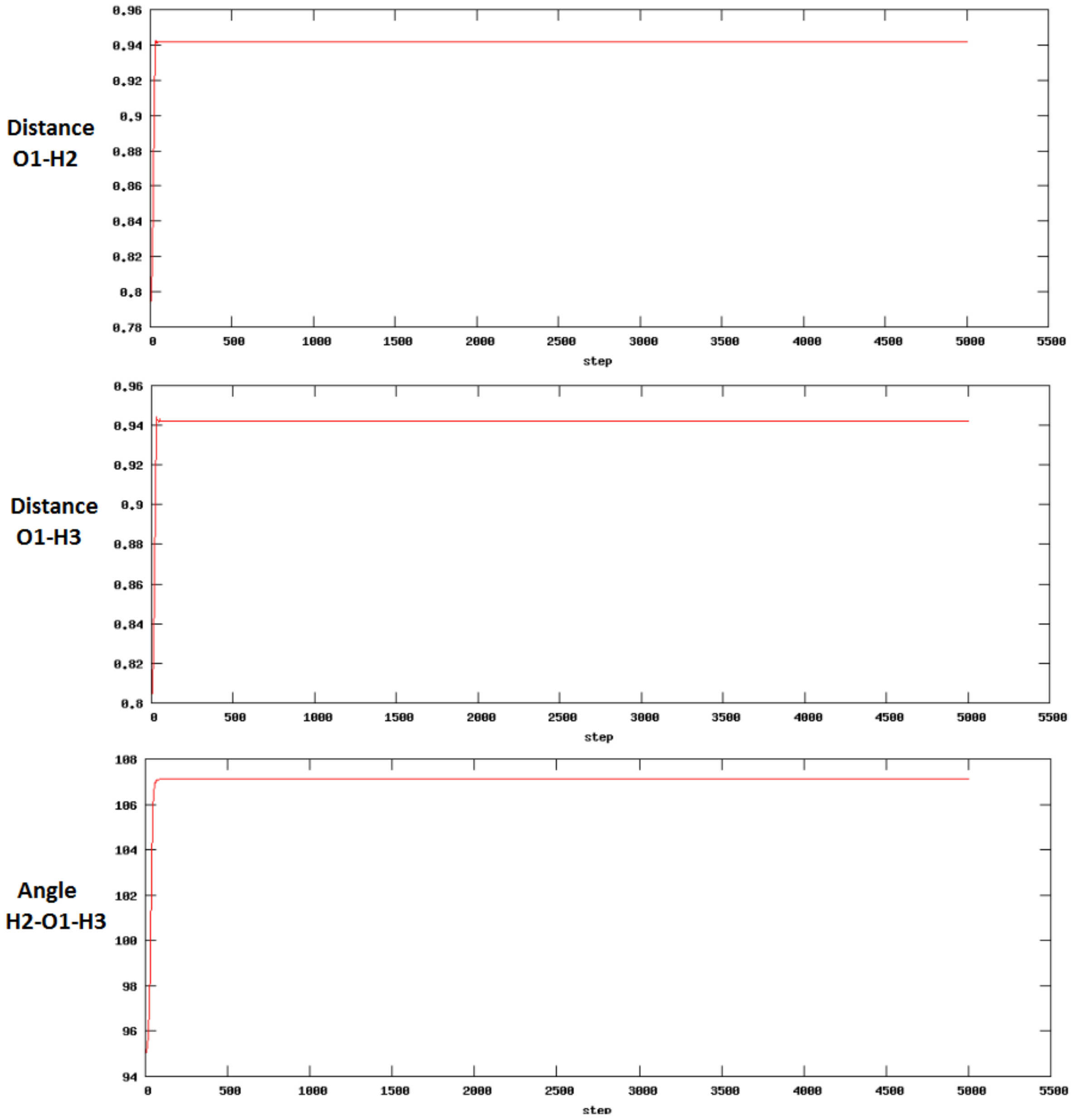

Figure S2. SP-OUT2 Set 1 with T500 model geometric feature optimization.

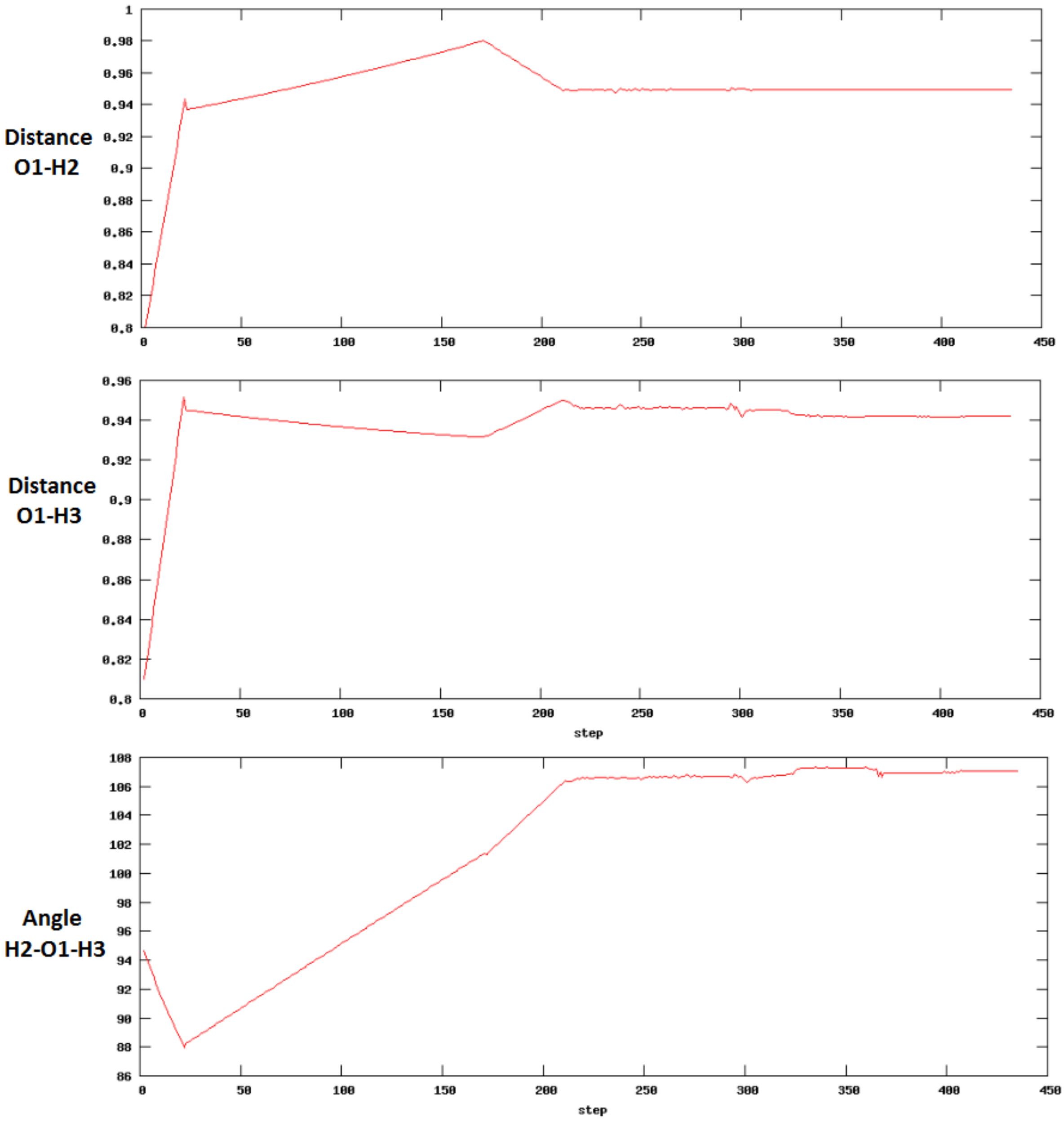

Figure S3. SP-OUT2 Set 4 with T500 model geometric feature optimization.

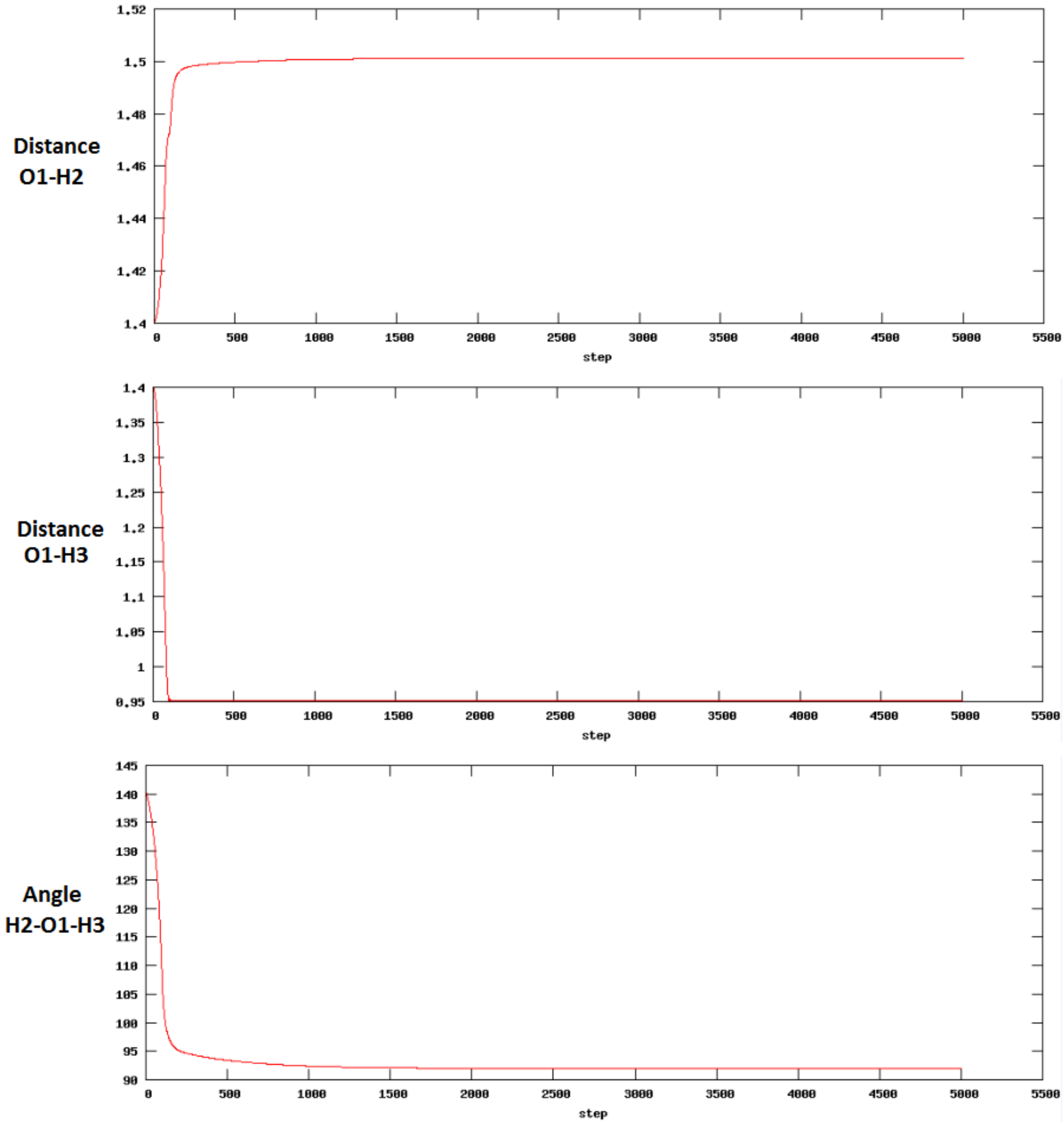

Figure S4. SP-OUT4 Set 1 with T500 model geometric feature optimization.

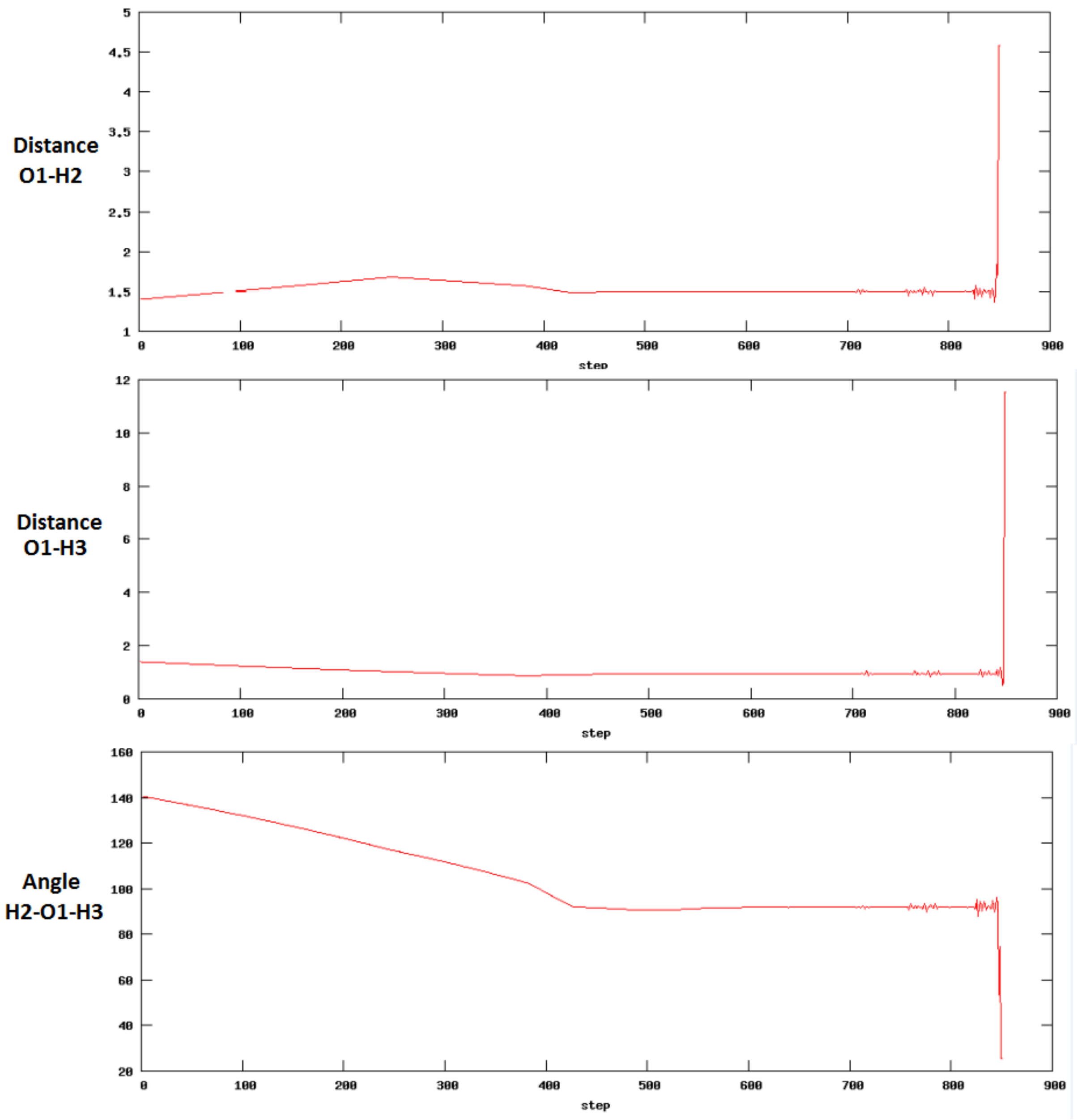

Figure S5. SP-OUT4 Set 4 with T500 model geometric feature optimization.
